# Exploring the asymmetric effect of COVID-19 pandemic news on the cryptocurrency market: evidence from nonlinear autoregressive distributed lag approach and frequency domain causality

**DOI:** 10.1186/s40854-022-00430-w

**Published:** 2023-01-13

**Authors:** Ştefan Cristian Gherghina, Liliana Nicoleta Simionescu

**Affiliations:** Department of Finance, Bucharest University of Economic Studies, 6 Romana Square, 010374 Bucharest, Romania

**Keywords:** COVID-19, Bitcoin, NARDL, EGARCH, Frequency domain causality

## Abstract

This paper explores the asymmetric effect of COVID-19 pandemic news, as measured by the coronavirus indices (Panic, Hype, Fake News, Sentiment, Infodemic, and Media Coverage), on the cryptocurrency market. Using daily data from January 2020 to September 2021 and the exponential generalized autoregressive conditional heteroskedasticity model, the results revealed that both adverse and optimistic news had the same effect on Bitcoin returns, indicating fear of missing out behavior does not prevail. Furthermore, when the nonlinear autoregressive distributed lag model is estimated, both positive and negative shocks in pandemic indices promote Bitcoin’s daily changes; thus, Bitcoin is resistant to the SARS-CoV-2 pandemic crisis and may serve as a hedge during market turmoil. The analysis of frequency domain causality supports a unidirectional causality running from the Coronavirus Fake News Index and Sentiment Index to Bitcoin returns, whereas daily fluctuations in the Bitcoin price Granger affect the Coronavirus Panic Index and the Hype Index. These findings may have significant policy implications for investors and governments because they highlight the importance of news during turbulent times. The empirical results indicate that pandemic news could significantly influence Bitcoin’s price.

## Introduction

The COVID-19 outbreak has generated a turbulent financial setting and set off a large-scale economic block that has driven a global recession (Naeem et al. 2021a). The significant sanitary slump caused by the pandemic has presented one of the most significant concerns since the Second World War (Khelifa et al. 2021). This unprecedented downturn was challenging to predict and is seen as a “black swan” event (Yarovaya et al. 2021). Initially, the coronavirus crisis impacted the real sector and provision of goods due to the interruption of industry and chain of distribution, extensive quarantines, and travel bans; afterward, the financial sector was sharply affected (Jebabli et al. 2021). Because of panic trading (Le et al. 2021), stock exchanges in the United States hit four circuit breakers in two weeks (Ji et al. 2020), and the oil price recorded a dramatic plunge, the largest since the Gulf War (Sharif et al. 2020). Due to the quick evolution of economic globalization, financial shocks may spread throughout nations and markets, and the global slowdown raises the likelihood of financial contagion (Guo et al. 2021). Hence, the integration of international financial markets has heightened considerably, and the market risk contagion between them has risen substantially (Liu et al. 2021). Furthermore, the market risk aversion amplified to levels not seen since the global financial crisis (Belhassine and Karamti 2021).

Destructive occurrences, such as COVID-19, engender fear among investors, which can modify their investment conduct, risk predilections, and, thus, asset prices (Papakyriakou et al. 2019). Béjaoui et al. (2021) reported short- and long-term support for the link between the Bitcoin price, social media metrics, and the intensity of the pandemic. Haroon and Rizvi (2020) found that panic induced by coronavirus news positively relates to volatilities in various manufacturing segments’ indices. Ambros et al. (2020) noticed that COVID-19 news boosted stock market volatility in European markets. In contrast, Sun et al. (2021) claimed that coronavirus-related news and economic-related announcements did not induce unreasonable investment resolutions. Various metrics that seize uncertain settings, including financial markets, were also used to predict future returns. In this regard, Costola et al. (2020) suggested that the Italian Google Trends (GT)-COVID-19 index account for other nations’ market returns. Bouri et al. (2021) observed that a daily newspaper-based index of equity market volatility due to infectious diseases enhances the prediction verity of gold realized variance at short-, medium, and long-run perspectives. Szczygielski et al. (2022) found that pandemic-related uncertainty, lockdowns, and media attention primarily impacted financial markets.

Cryptocurrencies are free of government manipulation (Gaies et al. 2021), but Bitcoin cannot ensure monetary stability, showing high volatility (Cachanosky, 2019; Anamika et al. 2021) and no fundamental value (Cheah and Fry 2015; Mnif et al. 2022). For instance, Easley et al. (2019) emphasized that the Bitcoin setting is shifting to a more market-based structure that can adjust to varying economic situations; however, the lack of regulation and transparency contributes to the market’s uncertainty (Wang 2021). Hence, like AlNemer et al. (2021), investor sentiment might exert a pivotal role in predicting Bitcoin price variations (Eom et al. 2019). Thus, Wołk (2020) emphasized that virtual currency price oscillations can rely on social media sentiment and web search analytics tools. This current study examines how Bitcoin returns react to COVID-19 pandemic news, measured by the RavenPack coronavirus-related indices (Panic, Hype, Fake News, Sentiment, Infodemic, and Media Coverage). The effects of the whole RavenPack pandemic indices on the cryptocurrency market has been underexplored. For instance, Chen et al. (2022) employed Panic Index, Media Hype Index, Fake News Index, and Sentiment Index, but the Bitcoin price ratio was considered instead of returns. Umar et al. (2021) used only the Media Coverage Index and established that virtual currencies are net shock transmitters, whereas fiat currency is a net receiver. Vurur (2021) employed only the Panic Index and found that digital coins are more vulnerable to adverse headlines. Umar and Gubareva (2020) covered only Coronavirus Panic Index but explored Bloomberg Galaxy Crypto Index. Marobhe (2022) covered the corona Panic Index and demonstrated the longstanding resistance of cryptocurrencies to COVID-19. As such, previous studies used only one indicator to assess media exposure, frequently overlooking the different influences of various types of news reports, such as disinformation and public anger (Zhang et al. 2022). In this regard, Atri et al. (2021) highlighted the heterogeneous impacts, documenting that COVID-19 panic has a negative effect on crude oil prices; however, COVID-19 media coverage has a positive influence on oil prices in the short-term. Furthermore, Shi and Ho (2021) noticed that pessimistic news tends to boost the probability of greater volatility states, whereas optimistic news has the opposite effect. Buigut and Kapar (2021) found a lowered (greater) exposure of panic and media indices (fake news) to the number of cases; however, this fact was not constant across all explored nations. Tan (2021) reinforced that media has varying degrees of influence across quantiles, and an asymmetric link exists between Borsa Istanbul returns and pandemic news. As such, a literature gap has been identified in documenting the effects of different pandemic news on Bitcoin; this gap in the literature motivated this study.

Certain events can arouse either a constructive or a pessimistic belief, substantially impacting investors’ investment preferences and, as a result, the related prices (Donadelli et al. 2017). In this regard, Jeon et al. (2021) supported that the regularity of the headline stream significantly impacts stock returns. Such reactions are primarily triggered by investors’ insights into news announcements and circumstances (Sun et al. 2021). Raissi and Missaoui (2015) suggest that investor sentiment is defined as a bias in which the response is based on a signal from noise rather than knowledge; it also refers to stockholder optimism or pessimism. Hudson et al. (2020) claimed that reasonable investment judgments are less likely to be made when anthropological, mental, and lifestyle factors enter the investment decision. Investigating how the cryptocurrency market reacts to coronavirus news is critical because it can assist investors and policymakers in making informed decisions. Our empirical findings support that positive and negative shocks in coronavirus-related indices boost Bitcoin returns. This notion is consistent with Salisu and Ogbonna (2021), which found a positive effect of media on the return instability of cryptocurrencies. Apart from providing evidence that pandemic news indices are a substantial driver of Bitcoin, our study also documents several causal associations between coronavirus-related indices and the cryptocurrency market.

Our main practical contributions to the existing literature are as follows. First, to the best of our knowledge, only Banerjee et al. (2022) approached the whole RavenPack pandemic indices on the cryptocurrency market; however, they did not consider any measure regarding new pandemic cases and deaths or the VIX index. Mahdi and Al−Abdulla (2022) used the RavenPack coronavirus news-based indices but did not include the Coronavirus Hype Index and Coronavirus Fake News Index. Youssef and Waked (2022) employed the Media Coverage Index but explored the herding behavior in the cryptocurrency market. Most of the previous studies employed Google (Urquhart 2018; Salisu and Ogbonna 2021; Anastasiou et al. 2021; Rajput et al. 2020; Figà−Talamanca and Patacca 2020; Zhu et al. 2021; Bashir and Kumar 2022; Vukovic et al. 2021; Benlagha and Hemrit 2022; Dias et al. 2022; Kim and Orlova 2021; Bonaparte and Bernile 2022; Raza et al. 2022b; Tong et al. 2022) or Twitter (Shen et al. 2019; Kraaijeveld and Smedt, 2020; Choi 2021; Naeem et al. 2020; Wu et al. 2021b; Elsayed et al. 2022; Bashir and Kumar, 2022; Kyriazis et al. 2022; Gök et al. 2022; Dias et al. 2022; French, 2021; Tong et al. 2022) data and were oriented toward equity markets (Haroon and Rizvi 2020; Shi and Ho, 2021; Tan 2021) or commodities (Atri et al. 2021). Because the effects of various kinds of news fluctuate, this paper employs different forms of COVID-19 pandemic news. Second, this study employs an extended period of investigation covering the first three destructive waves of the coronavirus pandemic. Third, previous research (Akyildirim et al. 2020; Corbet et al. 2020) considered the standard generalized autoregressive conditional heteroskedasticity (GARCH), which does not seize the uneven reactions of Bitcoin to positive and negative news. In contrast, this study employs the exponential GARCH (EGARCH) model, which permits the capture of volatility asymmetry (Tiwari et al. 2019). Furthermore, prior papers employed the robust least squares estimation method (Anamika et al. 2021), difference-in-difference framework (Chen et al. 2022), quantile regression model (Rahadian and Nurfitriani, 2022), or linear autoregressive distributed lag (ARDL) models (Demir et al. 2020; Vurur 2021; Havidz et al. 2022b) which do not consider the asymmetric effect. Therefore, we have used the nonlinear autoregressive distributed lag (NARDL) model of Shin et al. (2014) to simultaneously capture both asymmetric long and short-run relationships among variables (González et al. 2021; Long et al. 2021). The study of the uneven effect is necessary since the asymmetry in public opinion is considerable (Soroka 2006). Finally, different from earlier studies that applied the traditional Granger causality test (Guégan and Renault 2021; Sabah 2020; Béjaoui et al. 2021; Naeem et al. 2020, 2021b; Polat et al. 2022; Bourghelle et al. 2022; Burggraf et al. 2021; Zhu et al. 2021; French 2021; Hou et al. 2021), we assess the causal association among variables through frequency domain causality test developed by Breitung and Candelon (2006). This technique presumes a dynamic approach for causality examination because it is applied over several alternative frequencies.

According to theory, any increase in market insecurity generates a capital outflow from risky assets to safer ones (Burggraf et al. 2021). In this regard, our study’s theoretical contribution is illustrated by the validation of Demir et al. (2018); Demir et al. (2020). They suggest that Bitcoin can be regarded as a hedging instrument in times of extreme unpredictability. Furthermore, in line with Bouri et al. (2017), we show that Bitcoin reacts positively to uncertainty as measured through RavenPack coronavirus-related indices; however, our empirical outcomes oppose Baur and Dimpfl (2018), who claimed that after positive shocks, noise trading leads, while informed investors trade more after destructive shocks. Contrary to Güler (2021), the fear of missing out (FOMO) conduct of speculative and irrational investors does not prevail; emotions do not lead the Bitcoin market.

The rest of the paper is organized as follows. “Prior literature” section reviews earlier literature, “Quantitative framework” section describes the data and quantitative methodology, “Econometric findings” section presents our econometric outcomes, and “Concluding remarks and policy implications” section concludes.

## Prior literature

### On the diversifier, hedge, and safe heaven properties of cryptocurrencies during the COVID-19 pandemic

The rapid expansion of the pandemic exhibited the negative side of globalization and how severe a global overflow may be between nations (Zaremba et al. 2021). Accordingly, this circumstance has triggered a flight-to-quality phenomenon (Disli et al. 2021) wherein many stakeholders shifted from holding risky assets to perceived safe-haven assets (Park, 2022) to lessen risks, reduce losses, and shield the value of their portfolios (Diniz-Maganini et al. 2021). Under such conditions, cryptocurrencies are regularly mentioned when discussing potential safe-haven investments. A safe-haven asset should be unconnected or negatively associated with another asset during market stress or instability (Baur and Lucey 2010). Cryptocurrencies are a prevalent nominee for a safe-haven asset, regarded as the “new gold” (Klein et al. 2018) due to their autonomy from monetary policy, role as worth preservation, and poor association with common assets (Conlon and McGee 2020). Conlon et al. (2020) argued that safe-haven characteristics might differ globally, and Li et al. (2021a) showed that Bitcoin’s predictive power for different country equity indexes changes. Nevertheless, the evidence of Bitcoin’s potential to offer a safe-haven from instabilities in conventional markets is debatable. This may be due to the absence of a bear market in the historical sample before the pandemic; thus, the safe-haven premise was not assessed under critical market circumstances. Shahzad et al. (2022) argued that it is challenging to forecast Bitcoin’s future stability because of its short existence.

Therefore, the first area of study emphasizes Bitcoin’s hedging potential, safe-haven, and diverse investment qualities in the face of uncertainty. Table 1 summarizes the literature in this area. For instance, Trichilli and Abbes (2022) claimed that Bitcoin is a safe-haven asset that should be integrated with commodities and equities for stronger asset allocation and hedging efficiency before and during pandemic cycles. Compared to the S&P 500 Index, gold, and the US Dollar Index, Wang and Wang (2021) found that Bitcoin market efficiency is more resistant throughout the plague, supporting its safe-haven asset quality. Melki and Nefzi (2022) reported that Ethereum is the strongest safe-haven for commodities, while Ripple has performed as a hedge asset to the Forex market throughout the contagion. Jiang et al. (2021) additionally showed that Stellar and Bitcoin are outstanding alternatives to hedging assets for individual and institutional stockholders. Guzmán et al. (2021) found that declines in movement worldwide lifted the traded volume of Bitcoin throughout the pandemic. Baur et al. (2018) reported that Bitcoin is uncorrelated with conventional assets, such as stocks, bonds, and commodities, in regular periods and across financial meltdowns. Bouri et al. (2018) reinforced that Bitcoin can behave as a safe-haven against global financial stress from a medium-term standpoint, while Corbet et al. (2021) noticed an increased liquidity of the cryptocurrency market after the official declaration of the pandemic.

**Table 1:** Brief review of previous studies on the properties of cryptocurrencies as a hedge, safe haven, and diversifier during the COVID-19 disease outbreak

Author(s)	Period	Variables	Quantitative methods	Empirical outcomes
Allen (2022)	August 7, 2015–July 23, 2021	Bitcoin, Ethereum, S&P500 Index	Regression analysis, generalised measure of correlation, non-parametric copula	Bitcoin and Ethereum do not offer a powerful tool for portfolio diversification
Ali et al. (2022)	August 1, 2011–September 1, 2019	Bitcoin, Dow Jones World Emerging, Dow Jones World Islamic, FTSE4GOOD Global, gold, silver, MSCI World Energy, US Economic Policy Uncertainty	Multivariate Generalized Autoregressive Conditional Heteroscedastic-Dynamic Conditional Correlation (MGARCH-DCC), Continuous Wavelet Transforms (CWT)	Bitcoin ensures equivalent hedging potential to commodities like gold and silver with respect to the policy uncertainty
Al-Shboul et al. (2022)	September 8, 2015–February 21, 2021	Bitcoin, Litecoin, Ethereum, Tether, Ripple	Quantile VAR mode	While Bitcoin lost its role as a leading hedger during the downturn, Litecoin served as a central hedger and/or a value saver both during and before the pandemic
Balcilar et al. (2022)	October 2, 2017–May 20, 2022	Bitcoin, Cardano, Bitcoin Cash, Ethereum, Litecoin, USD Tether, Ripple, 27 emerging equity markets	Standard vector autoregressive (VAR) model, frequency decompositions of connectedness measures, quantile connectedness approach, lasso VAR	For emerging stock markets, major cryptocurrencies cannot be used as a diversifier
Bashir and Kumar (2022)	January 23, 2020–31 July 31, 2021	20 major cryptocurrencies based on market capitalisation	Simple linear regression, quantile regression (QR), the exponential generalised autoregressive conditional heteroskedasticity (EGARCH) model, sentiment analysis	Because cryptocurrencies do not behave autonomously during bear markets brought on by the COVID-19, they cannot serve as a safe haven
Będowska-Sójka and Kliber (2021)	August the 10, 2015–April 24, 2020	Bitcoin, Ether, S&P 500, DAX, FTSE250, STOXX Europe 600 Index, gold	Multivariate stochastic volatility model with dynamic conditional correlation	Neither Bitcoin nor Ether should be regarded as safe-haven assets
Cai et al. (2022)	January 2012–June 2021	Bitcoin price, economic policy uncertainty	Wavelet analysis	Although the Bitcoin market can be regarded as a prominent gauge, it cannot be treated as a safe-haven asset hedge against the economic policy uncertainty
Chemkha et al. (2021)	April 29, 2013–January 5, 2021	Bitcoin, gold, indices, exchange rates	Asymmetric dynamic conditional correlation (A-DCC) model	Bitcoin cannot ensure protection throughout the COVID-19 outbreak due to its considerable variation
Diniz-Maganini and Rasheed (2022)	July–December 2020	Bitcoin, MSCI	Detrended partial-cross-correlation analysis (DPCCA)	For the MSCI index, Bitcoin was never a safe haven
Dutta et al. (2020)	December 2014–March 2020	Bitcoin, WTI, Brent, Gold	DCC-GARCH	During the COVID-19 pandemic, Bitcoin only serves as a diversifier
Grira et al. (2022)	January 1, 2019–December 31, 2020	Bitcoin, S&P 500 index	Granger causality test, least squares (OLS) with the Newey-west estimator	During the COVID-19 crisis, Bitcoin can be viewed as a weak safe haven asset
Grobys (2021)	March 19, 2015–March 18, 2020	Bitcoin, S&P 500 index, gold	Difference-in-differences estimation	Bitcoin is not a reasonable tool for mitigating tail risk in US stocks
Hasan et al. (2022)	December 30, 2013–February 21, 2021	Bitcoin, gold, US Dollar index, Dow Jones World Islamic Market index, Dow Jones World Sukuk index, Crude Oil West Texas Intermediate, cryptocurrency policy uncertainty	Ordinary least squares (OLS), quantile regression (QR), quantile-on-quantile (QQ) regression	Bitcoin, US dollar, and WTI do not possess any safe-haven characteristics
Kumar and Padakandla (2022)	January 5, 2015–December 31, 2020	Bitcoin, Gold, DJIA, CAC40, NSE50, S&P 500, NASDAQ, EUROSTOXX	Wavelet Quantile Correlation	Bitcoin have shown long-term diversifier features but no safe haven characteristics for a highly market capitalized index like the S&P500
Maitra et al. (2022)	August 1, 2019,–May 29, 2020	Bitcoin, Ethereum, eight stock market indices	Copula-based VaR and CoVaR models	Cryptocurrencies are unable to generate additional earnings by reducing stock market risk in the face of the COVID-19 pandemic
Omane-Adjepong and Alagidede (2021)	January 5, 2015–August 11, 2020	Bitcoin, precious metals, Africa’s emerging equity markets	Two-stage DCC-GARCH	Bitcoin is not a superior safe haven alternative, only a complementary one
Rao et al. (2022)	August 2011–July 2021	Bitcoin, S&P Green Bond Index, S&P GSCI Crude Oil, S&P GSCI Gold Index, MSCI Emerging Markets Index, MSCI World Index	Time-varying parametric vector autoregression, quantile regression	Instead of acting as a hedge, cryptocurrencies behave as a safe haven for certain international indices at particular times
Ren et al. (2022)	January 23, 2020–August 19, 2020	Bitcoin, gold, WTI crude oil futures price, Brent crude oil futures	The varying-coefficient quantile regression	Oil-related portfolios have been found to benefit from Bitcoin’s role as a safe haven
Singh et al. (2022)	July 2016–June 2021	Bitcoin returns, economic policy uncertainty, geopolitical risk	Wavelet coherence analysis	In P5 + 1 countries, Bitcoin can be used as hedge against policy uncertainties and geopolitical risk
Syuhada et al. (2022)	September 29, 2018–March 31, 2021	Bitcoin, gold, energy commodities	Vine copula	The safe-haven feature of Bitcoin was regarded as unreasonable
Ustaoglu (2022)	August 7, 2011–November 16, 2021	Bitcoin, Ethereum, 27 main emerging market indices	Asymmetric dynamic conditional correlation-generalized autoregressive conditional heteroskedasticity model (ADCC-GARCH)	Bitcoin and Ethereum have safe-haven attributes relative to the most of emerging stock market indices during the pandemic timeframe
Vukovic et al. (2021)	January 22, 2020–April 11, 2020	Bitcoin, Ethereum, XRP, Tether, Bitcoin Cash, gold, crude oil, VIX, S&P 500 index	OLS (ordinary least squares), quantile, robust regressions	During COVID-19 crisis, the cryptocurrency market cannot serve as a haven or a hedge
Wen et al. (2022)	January 3, 2019–June 4, 2021	Bitcoin, COMEX gold futures price, the WTI oil price, the S&P 500 index	Time varying parameter vector auto-regression (TVP-VAR)	Bitcoin cannot be considered a safe haven
Yang et al. (2022)	January 15, 2015–December 31, 2015, and December 31, 2019–August 21, 2020	Bitcoin, gold, crude oil, commodities	Connectedness analysis, Wavelet-based DCC-GARCH model	In the long run, Bitcoin is relatively appropriate for hedging to lower portfolio volatility. It also performed well as a safe haven during the Swiss franc black swan event and phase I of COVID-19

*Source* Authors’ own work

Nonetheless, several studies questioned the hedge and safe-haven properties of Bitcoin. In this regard, Smales (2019) advised that Bitcoin is more unpredictable, less liquid, and more expensive to trade than gold, which should exclude it as a safe-haven asset. Furthermore, Choi and Shin (2022) found that Bitcoin prices diminish considerably due to financial uncertainty shocks. Raheem (2021) noticed that before the pandemic, Bitcoin held its broadly agreed qualities, but the post-COVID-19 proclamation revealed that the safe-haven assumption has faded. Cocco et al. (2022) reinforced this finding, advising that the pandemic has affected Bitcoin’s prestige as a safe-haven. Regarding the instability of returns, Yarovaya et al. (2022) claimed that virtual currencies depict the riskiest asset class in the long-term; hence, their safe-haven feature is questionable. Karaömer (2022) confirmed that throughout the prominent incidents, cryptocurrency policy uncertainty (UCRY Policy) has a detrimental effect on digital currency returns, suggesting that they are ineffective as a hedge or safe-haven asset. By investigating the initial equities bear market related to the pandemic, Conlon et al. (2020) reported that Bitcoin and Ethereum are not a safe-haven for most of the explored global equity markets because their integration increased portfolio downside risk. López−Cabarcos et al. (2021) suggested that Bitcoin can be considered a safe-haven during turbulent periods but is desirable for speculative investors during stable terms. Furthermore, Arouxet et al. (2022) also questioned the viability of cryptocurrencies as a safe-haven during the disease outbreak because they were vulnerable to speculative moves, and significant shifts in volatility could indicate the uncertainty surrounding the real price. Hence, the phases of obvious bubble behavior (Corbet et al. 2018) and the doubting dealing operation (Gandal et al. 2018) may cast skepticism on Bitcoin’s capability to serve as a safe-haven.

According to Huang et al. (2021), the COVID-19 outbreak changed the role of Bitcoin globally, except for the United States, which may account for the conflicting results regarding the hedge and safe-haven status of cryptocurrencies in the pandemic era. Furthermore, Wang et al. (2019) proved that the safe-haven feature is more noticeable in developed markets and subgroups with greater market capitalization and liquidity. In this regard, Wüstenfeld and Geldner (2022) argued that Bitcoin acts differently based on the nation being investigated, highlighting the significance of country-level studies.

### The effect of the COVID-19 pandemic on cryptocurrency returns 

Due to low fundamental value, Mnif et al. (2022) proved that the major cryptocurrency markets experienced several short-lived bubbles during the coronavirus pandemic. Market stress increased belief dispersion, decreasing Bitcoin futures returns but significantly elevating volatility and trading volume in the pandemic phase compared to the pre-pandemic period (Park 2022). In contrast to the S&P 500 Index and gold, which usually alternated between 3 and 5%, Foley et al. (2022) found that the expected risk premium for Bitcoin is considerably higher than other markets, averaging around 80% yearly. Foroutan and Lahmiri (2022) reinforced that cryptocurrencies are more erratic and unstable than global stock markets during the COVID-19 pandemic. Furthermore, Benlagha and Hemrit (2022) suggested that high stock market risk aversion and fear increase the value of Bitcoin; however, Haffar and Fur (2022) asserted that, except for a bear market, no asset could impact Bitcoin because it is a solitary market.

Furthermore, another branch of studies explored the relationship between coronavirus figures and digital asset returns, finding contradictory outcomes. Table 2 provides a summary of prior literature in this regard. Although the COVID-19 pandemic harmed the economy, Lee et al. (2022) showed that it had no discernible impact on Bitcoin; hence, some earlier research supported the conclusion that there is no connection between COVID-19 and the cryptocurrency market. In this regard, apart from USDT, Minutolo et al. (2022) noticed that the spread variation of the entire world has no impact on the price return of the major cryptocurrencies. Kim and Orlova (2021) estimated multivariate regressions, finding that the pandemic occurrence barely affected the performance of Bitcoin futures. Havidz et al. (2022a) uncovered that the COVID-19 cumulative positive cases had positive but insignificant effects on Bitcoin returns. Additionally, Vukovic et al. (2021) discovered that the COVID-19 crisis had no statistically significant direct impact on the cryptocurrency market during the initial wave, and Fernandes et al. (2022) demonstrated that cryptocurrencies displayed significantly stable price dynamics both before and during the pandemic. Furthermore, Fareed et al. (2022), among other studies, reported a nonlinear relationship between COVID-19 and Bitcoin. Hou et al. (2021) also found a short-term negative effect of COVID-19 on Bitcoin prices but a long-term beneficial effect due to its features, such as digital payments, unbanked assets, and safer virus propagation. Marobhe (2022) proved that Bitcoin, Ethereum, and Litecoin all experienced sizable negative return shocks during the first wave of COVID-19; however, they bounced back in April 2020 and remained resilient to subsequent COVID-19 panic shocks.

**Table 2:** Brief review of earlier studies on the nexus between COVID-19 outbreak and cryptocurrency market

Author(s)	Period	Variables	Quantitative methods	Empirical outcomes
Apergis (2021)	February 1, 2020–October 31, 2021	Bitcoin, Dash, Ethereum, Litecoin, XRP, NEM, DigiByte, Dogecoin, global established cases, global fatality cases	TGARCH, GJR-GARCH	The pandemic has a beneficial impact on the volatility of returns
Caferra and Vidal-Tomás (2021)	November 1, 2019–June 1, 2020	S&P 500, Euro Stoxx 50, Bitcoin, Ethereum	Wavelet coherence approach, Markov switching autoregressive model	COVID-19 caused a temporary effect on cryptocurrency dynamics
Du (2022)	January 11, 2019, to May 1, 20,201,	Bitcoin price, daily number of newly confirmed COVID-19 cases in China and in the United States	ARMAX, GARCH	Under the ARMAX model, there is no significant link between Bitcoin price and COVID-19, but the GARCH model exhibit a significant association
Iqbal et al. (2021)	January 1, 2020–June 15, 2020	Top ten cryptocurrencies ac- cording to market capitalization, regular additions in the active cases, everyday addition in number of deaths	QQR (Quantile-on-Quantile Regression)	Asymmetric effect of contagion severity on the downward and Bullish prognoses in cryptocurrencies
Jalan et al. (2021)	March 2020–August 2021	Digix Gold Token, Perth Mint Gold Token, Tether Gold, PAX Gold, Midas Touch Gold	Tail copula, dynamic spillovers	During the COVID-19 pandemic, the volatility of gold-backed cryptocurrencies was similar to that of Bitcoin
Keramiyan and Gokmenoglu (2022)	September 2010–June 2020	Bitcoin prices, Macroeconomic Uncertainty Index (MUI), Economic Uncertainty Related Queries (EURQ)	Conventional Granger causality, Granger causality test in quantiles	Bitcoin can act as a hedge against macroeconomic uncertainty during prolonged financial distress
Mariana et al. (2021)	July 1, 2019–April 6, 2020	Bitcoin, Ethereum, gold, S&P 500 daily returns	Dynamic conditional correlation analysis, regression analysis	Bitcoin and Ethereum exhibit large daily return volatilities throughout pandemic
Mgadmi et al. (2022)	January 2, 2019–July 27, 2021	Bitcoin, Ethereum, Stellar, Ripple, Cardano, cases, deaths and vaccination during the pandemic	ARMA(p,q), ARCH, GARCH, EGARCH, TGARCH, Ordinary Least squares method	Except for Cardano, the overall death toll has a negative effect on cryptocurrencies’ price The overall population with the disease and the total population who have received vaccinations have a positive impact on the cryptocurrency market
Mnif et al. (2020)	April 29, 2013–May 19, 2020	Bitcoin, Ethereum, Ripple, Litecoin, Binance	Multifractal analysis	COVID-19 improved the efficiency of the digital currencies
Raza et al. (2022a)	January 19, 2020–April 26, 2021	Ethereum, Stellar, Bitcoin, Ripple, Binance Coin, Litecoin, Cardano, Chain Link	Time-varying parameter vector autoregressions (TVP-VAR), causality-in-quantiles	The spillover connectedness across the virtual currencies is significantly impacted by COVID-19
Sahoo (2021)	March 10, 2020–June 30, 2020	Bitcoin, Ethereum, Bitcoin Cash, Ripple, Litecoin, COVID-19 established and death cases	Linear and nonlinear Granger causality	Unidirectional causal relation from COVID-19 figures to cryptocurrency price returns
Sahoo and Rath (2022)	March 15, 2020–December 15, 2021	Bitcoin, total number of confirmed cases and total number of deaths caused by the COVID-19	Frequency-domain granger causality	The association between the overall number of reported COVID-19 cases and Bitcoin returns was only ascertained at short and medium frequency bands
Vidal-Tomás (2021)	August 1, 2019–August 1, 2020	69 cryptocurrencies	Network analysis	The virtual currency market was impacted by COVID-19 on March 12, 2020, but the market has gradually rebounded to its original conditions since April 2020
Wasiuzzaman and Rahman (2021)	October 2, 2019–September 28, 2020	Gold-backed cryptocurrencies	ARMA-GARCH model	The average yields and volatility for both PAX Gold and Gold are larger during the pandemic and bear market stages, but the effect is non-significant
Yan et al. (2022)	September 8, 2017–February 14, 2022	Ten cryptocurrencies	Generalized auto-regressive conditional heteroscedasticity (GARCH) model, dynamic conditional correlation (DCC)	COVID-19 had a beneficial impact on crypto returns

*Source* Authors’ own work

By examining the closing prices of Bitcoin, Ripple, Litecoin, and Dash, Nitithumbundit and Chan (2022) reported greater return persistence, volatility, and cross-dependency during the disease outbreak, proving increased risk. Furthermore, Usman and Nduka (2022) observed a rise in persistence levels compared to before COVID-19 was declared a pandemic. Sui et al. (2022) confirmed that the cryptocurrency market was impacted by COVID-19, which substantially increased its total risk spillover effect. Similarly, Nguyen (2022) confirmed a volatility spillover effect from the stock market to Bitcoin throughout the pandemic phase and other times of extreme uncertainty. Abraham (2021) used an event study approach and noticed that around COVID-19 dates, both Bitcoin and Altcoins experienced negative abnormal returns, with Altcoins being more adversely impacted. According to Bashir and Kumar (2022), a 1% rise in the Google search volume index, Twitter economic uncertainty, and tweets leads to a reduction in Bitcoin returns of 0.44, 0.33, and 1.35%, respectively. Demir et al. (2020) identified a negative connection among Bitcoin value and the number of reported cases and deaths; however, the relationship turns into positive throughout the subsequent period. In this regard, Di and Xu (2022) argued that despite the increasing number of new COVID-19 cases brought on by Omicron, the vaccine boosted confidence and sped up the financial market recovery, significantly positively impacting the ability of the financial market to recover from the pandemic.

Other research suggested that the pandemic had a beneficial impact on cryptocurrency returns. According to Mzoughi et al. (2022), the performance of the digital gold-containing portfolio improved during the COVID-19 crisis, particularly in cumulative returns. Temkeng and Fofack (2021) noticed that new COVID-19 deaths strongly impacted the price of cryptocurrencies, but not by new confirmed cases, total cases, or total deaths. Goodell and Goutte (2021) confirmed that levels of COVID-19 instigated an increase in Bitcoin prices. Karamti and Belhassine (2021) found that the more US-COVID-19 fear increases, the more investors run to Bitcoin. Furthermore, Sarkodie et al. (2022) documented a mean daily surge in the market price of Ethereum, Bitcoin, Litecoin, and Bitcoin Cash by 0.58%, 0.44%, 0.36%, and 0.15%, respectively, when COVID-19 confirmed cases and deaths rose by 3.77%, and 3.65% daily. Similarly, Corbet et al. (2020) reported a significant increase in both returns and trading volumes, implying that sizable virtual currencies functioned as a store of wealth throughout this period of intense financial market tension.

### The nexus between pandemic news and the cryptocurrency market

The increasing prevalence of the COVID-19 pandemic heightened pessimism in the world’s leading markets (Dash and Maitra 2022). An economic individual does not constantly act reasonably because their judgments are altered by beliefs (Huynh et al. 2021). Poor tempers and distress may influence investor choices, such as tense individuals losing hope concerning upcoming returns, inclining them to take less risk (Kaplanski and Levy 2010). Positive feedback trading or trend chasing implies that investors buy securities when prices increase and sell when prices go down (Long et al. 1990), while negative feedback or contrarian trading implies buying after price decline (Cutler et al. 1990). King and Koutmos (2021) identified a discrepancy in trading design, namely trend chasing for Bitcoin, Ethereum, XRP, and Cardano, but contrarian trading for EOS and Stellar; hence, Agosto et al. (2022) proved that sentiment is crucial in early warning bubble signals.

Cryptocurrencies are renowned for their extreme volatility and long-term fluctuations brought on by investors’ emotions; they are not traded on regulated markets and are not subject to the same regulations as traditional financial instruments (Assaf et al. 2022). Sentiment analysis is a widely researched field in the era of social media and has been employed to boost trading cryptocurrency estimations (Fang et al. 2022). Thus, the cryptocurrency market heavily mirrors media platforms, with high aspirations, quick swings in sentiment, definite opinions, and intense debates (Aste, 2019). Specifically, the use of emotion statistics obtained via social media and based on a glossary of words allows for evaluating opinions depending on the severity of the pandemic and the interconnections between such feelings and cryptocurrencies (Corbet et al. 2020). Bowden and Gemayel (2022) evidenced that emotion influences investors’ decisions because bullish sentiment generates positive returns for cryptocurrency traders. Umar and Gubareva (2020) claimed that cryptocurrency markets are highly responsive to overall sentiment and vulnerable to mainline anticipations, particularly throughout crises such as the COVID-19 pandemic. The third strand of literature is oriented on how investor sentiments extracted from news, social networks such as Twitter, or investor attention from Google influence Bitcoin. Urquhart (2018) found that Google Trends, as a measure of investor attention, is affected by Bitcoin’s previous day high realized volatility and volume. Shen et al. (2019) proved that the number of tweets on Twitter significantly drives Bitcoin’s future realized volatility and trading volume. Kraaijeveld and Smedt (2020) confirmed that Twitter sentiment can be used to forecast the price returns of Bitcoin, Bitcoin Cash, and Litecoin, while Naeem et al. (2020) showed that Twitter Happiness Index is a significant predictor of Bitcoin, Ethereum, Ripple, Litecoin, and Monero, contingent on the market status. Huynh (2021) noticed that more pessimistic Trump sentiments led to higher Bitcoin returns. Choi (2021) exhibited that the number of tweets positively influences Bitcoin liquidity. Contrariwise, Anastasiou et al. (2021) exhibited that investors’ crisis sentiment proxied by the Financial and Economic Attitudes Revealed by Search index positively influences cryptocurrencies’ market price crash risk. Besides, Sifat (2021) supported the detachment of cryptocurrencies’ price, volatility, and trading operations from global sentiments over 2015−2021. Table 3 presents a brief review of the literature in this direction.

**Table 3:** Summary of prior literature on Bitcoin reaction to investors’ sentiment and various news

Author(s)	Period	Variables	Quantitative methods	Empirical outcomes
Aharon et al. (2020)	January 1, 2011–July 4, 2020	Bitcoin, Ethereum, Bitcoin-cash, Ripple, Twitter Market Uncertainty Index, Twitter Economic Uncertainty Index	OLS, GARCH, Granger-causality in distributions	Strong causal connection among the social media uncertainty and cryptocurrency returns
AlNemer et al. (2021)	January 15, 2013–November 15, 2020	Bitcoin, Dogecoin, Ethereum, Litecoin, Tether, Sentix Investor Confidence	Wavelet coherency analysis	Long-term positive connection between Bitcoin prices and Sentix Investor Confidence
Aslanidis et al. (2022)	August 7, 2015–April 22, 2021	Bitcoin, Google Trends Cryptocurrency Attention Index (GTC)	Transfer entropy	Two-way stream of information among GTC and cryptocurrency returns up to six days
Banerjee et al. (2022)	January 1, 2020–April 15 2021	Top 30 cryptocurrencies by market capitalization, RavenPack COVID-19 sentiments	Transfer entropy	The connection between COVID-19 news sentiment and cryptocurrency returns is nonlinear
Bonaparte and Bernile (2022)	January 2004 –March 2022	Bitcoin, Ethereum, BNB, Cardano, Solano, Terra, Dogecoin, Crypto Regulation Sentiment Index (CRSX)	Regression analysis	There is no statistically significant long-term effect of CRSX on the price of cryptocurrencies
Bourghelle et al. (2022)	January 21, 2020–May 25, 2021	Bitcoin, fear and greed index	Linear and nonlinear vector autoregressive (VAR) model	The impact of market sentiment depends on time
Bouteska et al. (2022)	January 1, 2015–September 30, 2022	Bitcoin, Cryptocurrency Index (CRIX), Volatility CryptoIndeX (VCRIX), sentiment measures based on StockTwits and Reddit	Principal component analysis (PCA) method, vector autoregressive model (VAR)	Investor sentiment, as assessed by messages pertaining to the financial aspect of cryptocurrencies, has a greater predictive ability and yields better outcomes than the cryptocurrency index, particularly during times of market turmoil
Burggraf et al. (2021)	April 2013–February 2019	Logarithmic Bitcoin returns, microeconomic and macroeconomic financial and economic attitudes revealed by search (FEARS)	Transfer entropy, threshold regression, ordinary least squares (OLS), generalized least squares (GLS), two-stage least squares (2SLS) regressions, VAR-Granger analysis	The effect of investor emotion on Bitcoin return is adverse and statistically significant
Chen et al. (2020)	January 15, 2020–April 24, 2020	Bitcoin price dynamics, VIX, Google Trends	Vector autoregressive (VAR) models	Growing fear of the coronavirus leads to negative Bitcoin returns and high trading volume
Ciaian et al. (2016)	November 2009 –May 2015	Bitcoin price, the volume of daily Bitcoin views on Wikipedia, new members and new posts on online Bitcoin forums	Vector error correction (VECM) model	The arrival of fresh news positively influence Bitcoin price
Dias et al. (2022)	January 1, 2017–December 31, 2021,	Bitcoin, Google search volume, Twitter happiness index, Wikipedia page views, news sentiment, VIX, daily merits shared in bitcointalk.org	Principal component analysis, Quantile regression approach	Bitcoin returns can be significantly predicted by investor interest and sentiment
Fang et al. (2020)	May 2013–May 2019	Bitcoin, Ethereum, Ripple, Litecoin, New Economy Movement, Global Economic Policy Uncertainty (GEPU), of News-based Implied Volatility (NVIX)	GARCH-MIDAS	Negative impact of NVIX on the cryptocurrencies’ long-term volatility
Figà-Talamanca and Patacca (2020)	January 2012–December 2018	Bitcoin returns and trading volume, Google Search Volume Index (SVI)	Vector AutoRegressive (VAR) model	Bitcoin returns are not affected by trading volume and SVI
French (2021)	October 1, 2013–September 15, 2020	Bitcoin, Twitter-based market uncertainty index	Pairwise Granger causality, Bayesian vector auto-regression (BVAR)	A significant predictor of Bitcoin returns only throughout the COVID-19 period is the Twitter-based market uncertainty index
Gaies et al. (2021)	August 2011–July 2020	Bitcoin Misery Index, VIX, the Kansas City Financial Stress Index, the 10-year US nominal interest rate	Nonlinear autoregressive distributed lag model	An increase in the level of optimistic (pessimistic) sentiment has a positive (adverse) influence on Bitcoin returns
Gök et al. (2022)	June 1, 2011–August 30, 2021	Bitcoin, gold, US10 year Treasury notes, Twitter-based economic uncertainty index, geopolitical risk index, US VIX, daily infectious disease equity market volatility tracker	Causality-in-quantiles, Wavelet decomposition	Causality-in-variance from Twitter-based economic uncertainty to Bitcoin
Guégan and Renault (2021)	August 2017–December 2019	Bitcoin prices and retuns, StockTwits sentiment	Multivariate regressions, Granger causality tests	Investor sentiment predict Bitcoin returns for high frequencies (up to 15 min)
Güler (2021)	February 2018–August 2020	Bitcoin trading volume, Crypto Fear & Greed Index, Weekly American Association of Individual Investors Index (AAII)	GARCH models, Vector autoregressive (VAR) model	Both rational and irrational investor sentiments influence Bitcoin returns
Havidz et al. (2022b)	March 18, 2021–August 31, 2021	Bitcoin, Ethereum, Vaccine confidence index, Global fear index, Panic index, Sentiment index, blockchain features	Autoregressive distributed lag (ARDL)	Negative connection among Global fear index and Bitcoin returns Vaccine confidence index and Global fear index were insignificant to Ethereum returns Panic index and Sentiment index were insignificant in the long run to Bitcoin and Ethereum returns
Jin et al. (2021)	July 9, 2012–June 24, 2013	Daily Bitcoin price	Empirical mode decomposition with adaptive noise (CEEMDAN)-based event analysis	The announcement of 2013 Cyprus bailout substantially intensified the strength of short-term oscillations in Bitcoin prices
Kim et al. (2022)	November 2017–April 2018 December 2018–May 2019	Trading volume and closing price of Bitcoin, number of Bitcoin searches on Google, number of positive/negative sentiments about Bitcoin	Hidden Markov model (HMM)	Optimistic social sentiment is more relevant throughout a bullish trend, whilst adverse social emotion is more significant during a bearish market
Kyriazis et al. (2022)	January 1, 2020–July 25, 2021	Bitcoin, Ethereum, Binance Coin, Cardano, Ripple, Dogecoin, Bitcoin Cash, Litecoin, Ethereum Classic, Stellar, economic and market uncertainty indices	Linear and nonlinear Granger causality tests	Twitter sentiment is noticed to have a significant impact on investigated cryptocurrencies
Li et al. (2022)	January 2012–October 2021	Bitcoin returns, Google News	Vector autoregressive (VAR) framework	In the bubble period, media coverage (whether positive or negative) has a positive link with Bitcoin returns the following day, but there is no significant relation in the post-bubble period
Li et al. (2021b)	January 2013–April 2019	Bitcoin return, domestic and foreign events	GARCH-X model	Domestic events positively influence Bitcoin price volatility, whereas foreign events impact both BTC price return and volatility
Lyócsa et al. (2020)	January 2013–December 2018	Bitcoin, news about the regulation of Bitcoin, hacking attacks on Bitcoin exchanges, investor sentiment, macroeconomic news	Quantile regressions	Increased Bitcoin volatility a day ahead of publication an article towards Bitcoin regulation
Mahdi and Al-Abdulla (2022)	January 3, 2020 −September 1, 2021	Bitcoin, gold, RavenPack coronavirus news-based indices	Quantile-on-quantile regression model	The distribution of Bitcoin returns is affected asymmetrically by positive and negative shocks in coronavirus-related news
Mai et al. (2018)	January 1, 2012–December 31, 2014	Bitcoin price, returns, trading and transaction volume, number of positive and negative posts, number of positive and negative tweets	Vector error correction (VECM) model	Social media sentiment can explain and predict Bitcoin value
Mokni et al. (2022)	January 2, 2018–December 10, 2020	Bitcoin, the fear and greed index	Symmetric and asymmetric causality analysis, quantile autoregressive regression model	The Bitcoin price has a strong impact on investor sentiment
Naeem et al. (2021b)	March 7, 2016–December 29, 2019	Bitcoin, Litecoin, Ripple, Dash, Monero, Ethereum, Twitter Happiness index, FEARS index	OLS, quantile regression (QR), cross-quantilogram (CQ)	Cryptocurrency returns are determined more by sentiment spread over social media than with macroeconomic news
Nair (2021)	September 1, 2018–April 30, 2021	Bitcoin, Ethereum, Litecoin, Neocoin	Decomposition of returns, GARCH framework, vector autoregressive model	The response of crypto markets to negative news is equivalent to how they respond to good news
Philippas et al. (2019)	January 1, 2016–May 28, 2018	Bitcoin prices, Twitter and Google Trends	Dual process diffusion model	Media networks have only a limited impact on Bitcoin prices, which is larger on periods with greater incertitude
Polat et al. (2022)	January 1, 2019 –January 31, 2021	Bitcoin, Thomson Reuters MarketPsych Indices	Bivariate vector autoregressive (VAR) models	A rise in fear sentiment has a longer and more significant adverse effect on Bitcoin returns
Rajput et al. (2020)	January 2013–December 2018	Bitcoin sentient index, Bitcoin returns, volume traded and volatility	Linear and nonlinear autoregressive distributed lag (ARDL) models	Positive connection of Bitcoin sentiment index with its returns and volume, but a negative connection with its return volatility
Raza et al. (2022b)	January 2016–March 2021	Bitcoin, Dash, Ethereum, Litecoin, NEM, Ripple, Google Trends data	Causality-in- quantiles test	The price of cryptocurrencies can be accurately predicted by using Google Trends
Rognone et al. (2020)	January 1, 2012–November 1, 2018	Bitcoin, Forex, Sentiment indices	Exogenous vector autoregressive (VAR-X) model	Bitcoin reacts positively to both positive and negative news, but cyber-attack and fraud news lessen its returns and volatility
Sabah (2020)	February 9, 2014–December 31, 2018	Venues that accept cryptocurrencies as a payment method, market capitalization and market cap Weighted Cryptoz Index Volatility for top 10, 25, 50 and 100 cryptocurrencies	Regression analysis, bivariate vector autoregression, Granger causality	Investor attention as measured through the number of new business venues that accept cryptocurrencies as a form of payment is a predictor of crypto volatility
Salisu and Ogbonna (2021)	September 2, 2019–September 29, 2020	Gtrend, Bitcoin, Ethereum, Litecoin, Ripple	GARCH-MIDAS	Fear-generated news set off by the COVID-19 pandemic boosts the return volatilities of the cryptocurrencies contrasted with the period prior to the contagion
Tong et al. (2022)	January 1, 2017–January 26, 2022	24 cryptocurrencies, Google Trends, daily numbers of Twitter tweets	Transfer entropy	Twitter has a higher information flow toward cryptocurrencies than the other way around
Vurur (2021)	January 8, 2020–December 31, 2020	Bitcoin, Ethereum, Ripple, Panic index	Autoregressive-Distributed Lag Cointegration, Hatemi-J asymmetric causality	Rises in the Panic index diminish the cryptocurrencies’ value
Wu et al. (2021b)	August 9, 2015–July 7, 2020	Bitcoin, Ethereum, Litecoin, Ripple, Twitter-based economic uncertainty and Twitter-based market uncertainty	Granger causality test using the recursive evolving window approach	Variations in the Twitter-based economic policy uncertainty (EPU) indices are positively connected to the cryptocurrencies’ returns during the COVID-19 period
Xia et al. (2022)	September 19, 2014–May 20, 2022	Bitcoin, Economic Policy Uncertainty (EPU) and Cryptocurrency Uncertainty (UCRY) indices	GARCH-MIDAS	Global economic policy uncertainty has a significant adverse impact on the long-term volatility of Bitcoin, whereas cryptocurrency uncertainty has a beneficial effect
Zhang et al. (2022)	January 1, 2020–September 18, 2020	Crude oil, gold, Bitcoin, RavenPack specific COVID-19 news-related indices	Time–frequency analysis method	Panic sentiment and media hype influence Bitcoin

*Source* Authors’ own work

National uncertainty is essential in Bitcoin investors’ decision-making since most of its related trading volume and holders are condensed in a few nations (Wu et al. 2021a). Elsayed et al. (2022) noticed that the volatility spillover of Bitcoin is driven by Economic Policy Uncertainty (EPU), but Twitter-based Economic Uncertainty does influence Bitcoin’s volatility. Shaikh (2020) also confirmed a negative relationship between uncertainty in the equity market and Bitcoin returns, whereas EPU influences Bitcoin returns. Yen and Cheng (2021) documented that a variation in the China EPU is negatively connected with the future volatility of Bitcoin and Litecoin. Conversely, Cheng and Yen (2020) claimed that China’s EPU index predicts Bitcoin returns, but the US, Japanese, and Korean EPU indexes do not. Mokni (2021) noticed that EPU could forecast volatility merely when the Bitcoin market is bullish.

Behavioral finance has exposed a range of preconceptions that affect investment judgments (Shrotryia and Kalra, 2021). Banerjee (1992) argued that individuals would follow others instead of utilizing their knowledge. Youssef and Waked (2022) proved that media could influence investors’ behavior regarding the coronavirus, causing them to ignore their personal information and replicate other people’s investment choices. As such, Jia et al. (2022) asserted that investor sentiment and herding behaviors are connected. According to Sapkota (2022), emotions have a medium-term effect on Bitcoin fluctuation, while financial viewpoints have a long-term influence. For instance, Wang et al. (2022) found that throughout the COVID-19 pandemic, market insecurity triggered by contagious illnesses contributes to a positive feedback trading mentality. Hence, the fourth stream of literature focuses on investors’ different biases. Gurdgiev and O’Loughlin (2020) noticed a surge in cryptocurrency prices when there prevails positivity among investors, thus suggesting the occurrence of herding biases. Mandaci and Cagli (2021) detected intensified herding conduct during the coronavirus outbreak, whereas Rubbaniy et al. (2021) confirmed herd investing after the relaxation of the isolation measures. According to Ferreruela and Mallor (2021), during the COVID-19 disease outbreak, herding is evidenced on days with high volatility. Furthermore, Kyriazis (2020) highlighted that bull markets can cause more severe herding than bear markets, contributing to biases. Anamika and Subramaniam (2022) supported that the cryptocurrency market exhibits herding conduct when the sentiment of investors is upbeat or bullish, which increases prices. Kakinaka and Umeno (2021a) claimed that COVID-19 enhanced herding in the short-term, but not in the long-term; however, Mnif and Jarboui (2021) claimed that the pandemic has lessened the herd bias. Moreover, Güler (2021) highlighted the FOMO behavior illustrated by the fear a Bitcoin investor encounters when overlooking a potentially profitable investment or trading opportunity.

Several features emerge from the preceding literature. First, there has been a heated debate about cryptocurrencies’ diversifier, hedge, and safe heaven qualities. Second, the literature regarding the impact of severe acute respiratory syndrome coronavirus 2 (SARS-CoV-2) on cryptocurrency returns reported conflicting results. Third, various proxies for investor sentiment proved to be significant drivers of Bitcoin price. Finally, most research findings reported an expansion of herding behavior throughout the pandemic period.

## Quantitative framework

### Data and variables

Our dataset consists of daily Bitcoin returns, COVID-19-related news measures, coronavirus figures, and the VIX index from January 2020 to September 2021. Table 4 shows the definitions of the whole covered variables. We selected Bitcoin as a proxy for the cryptocurrency market since it is the primary, largest-capped (Raza et al. 2022b), and most prominent virtual currency (Anamika et al. 2021; Tiwari et al. 2019), recognized as a substitute payment method by many traders (Feng et al. 2018; Burggraf et al. 2021; Diaconaşu et al. 2022; Karaömer, 2022). According to Sebastião and Godinho (2021), Bitcoin’s “ecosystem” possesses many characteristics, including immateriality, decentralization, accessibility, and consensualness. It is also integer-based, transparent, worldwide, quick, affordable, irreversible, immutable, divisible, resilient, and pseudonymous. Bitcoin’s underlying technology, blockchain, also has several benefits, including distributed ledger, decentralization, information transparency, tamper-proof design, and openness (Xu et al. 2019). In line with Chen et al. (2022), Mahdi and Al–Abdulla (2022), and Banerjee et al. (2022), we obtained coronavirus indices from RavenPack. RavenPack Coronavirus Media Monitor synthesizes the feelings (mood) expressed in news reports and public posts, which are then converted into convenient metrics (Rahadian and Nurfitriani 2022). The Coronavirus Panic Index is calculated by dividing the daily count of unique stories concerning panic key phrases and coronavirus by the daily number of distinct stories referencing panic search terms and coronavirus. The Coronavirus Hype Index is estimated by dividing the regular count of distinct stories citing the coronavirus by the total daily count of unique stories. The justification is that the more individuals are subjected to intensified media hysteria about COVID-19, the more depressed they may feel about the economy and the more they favor digital currencies (Chen et al. 2022). The Coronavirus Fake News Index is calculated by dividing the daily count of different stories that cite false information and the coronavirus by the total daily count of unique articles. The Coronavirus Sentiment Index is the ratio between the daily median of the RavenPack’s Event Sentiment Score (ESS) for all identified headlines concerning the coronavirus and the everyday median of the ESS for all occurrences that do not cite the coronavirus. This disparity is then averaged over the previous seven calendar days. The Coronavirus Infodemic Index is obtained by dividing the regular number of distinct entities referenced with the coronavirus by the total daily count of unique entities. The Coronavirus Media Coverage Index is figured by dividing the total daily count of distinct media sources that notice the coronavirus by the everyday total number of distinct news outlets that cite the coronavirus.

**Table 4:** Variables’ description

Variable	Definition	Source
BTC	Daily changes of Bitcoin price (BTC/USD – Bitcoin US Dollar)	Investing.com
PI	The Coronavirus Panic Index – measures the level of news chatter that makes reference to panic or hysteria alongside the Coronavirus. Values range between 0 and 100 where a value of 7.00 indicates that 7 percent of all news globally is talking about panic related terms and COVID-19	RavenPack
HI	The Coronavirus Hype Index measures the percentage of news talking about the novel Coronavirus. Values range between 0 and 100 where a value of 75.00 indicates that 75 percent of all news globally is talking about COVID-19	RavenPack
FNI	The Coronavirus Fake News Index measures the level of media chatter about the novel virus that makes reference to misinformation or fake news alongside COVID-19. Values range between 0 and 100 where a value of 2.00 indicates that 2 percent of all news globally is talking about fake news and COVID-19	RavenPack
SI	The Coronavirus Sentiment Index measures the level of sentiment across all entities mentioned in the news alongside the Coronavirus. The index ranges between -100 and 100 where a value of 100 is the most positive sentiment, −100 is the most negative, and 0 is neutral	RavenPack
II	The Coronavirus Infodemic Index calculates the percentage of all entities that are reported in the media alongside COVID-19. Values range between 0 and 100 where a value of 60.00 means that 60 percent of all entities covered by the media are being co-mentioned with COVID-19	RavenPack
MCI	The Coronavirus Media Coverage Index calculates the percentage of all news sources covering the topic of the novel Coronavirus. Values range between 0 and 100 where a value of 60.00 means that 60 percent of all sampled news providers are currently covering stories about COVID-19	RavenPack
CNC	Daily number of new reported COVID-19 cases worldwide (logarithmic values)	Our World in Data
CND	Daily number of new reported COVID-19 deaths worldwide (logarithmic values)	Our World in Data
VIX	Daily change of Chicago Board Options Exchange (CBOE) volatility index	Investing.com

Source: Authors’ own work

The quotidian number of novel pandemic cases and fatalities globally was covered in line with Atri et al. (2021), Buigut and Kapar (2021), Béjaoui et al. (2021), Iqbal et al. (2021), Apergis (2021); Sahoo (2021), Chen et al. (2022), Hou et al. (2021), Sahoo and Rath (2022), Havidz et al. (2022a), and Temkeng and Fofack (2021). According to Trichilli and Abbes (2022), COVID-19 data serve as a valuable device permitting forecasting of returns of cryptocurrencies, commodities, and stock markets. Following the extant literature, the VIX index was included to measure US market uncertainty (Sabah 2020; López–Cabarcos et al. 2021; Gaies et al. 2021; Chen et al. 2020; Smales, 2022; Anamika et al. 2021; Akyildirim et al. 2020; Gök et al. 2022; Dias et al. 2022; Minutolo et al. 2022). Su et al. (2022) considered that Bitcoin oscillations might be influenced by market concern, as assessed by the VIX, while Elsayed et al. (2022) found that Bitcoin usually received returns spillover from the VIX. Bouri et al. (2017) argued that greater values of the VIX imply more market insecurity and vice-versa. According to Smales (2022), volatility in US markets is critical to worldwide stock market insecurity, even if fluctuations in international market uncertainty do not explain shifts in US market turmoil. Furthermore, because the VIX information is a valuable resource for stockholders, it is essential to incorporate the VIX in any study of Bitcoin’s power to hedge or its connection with other assets (Bouri et al. 2017).

### Empirical methods

#### Asymmetric GARCH framework toward cryptocurrency market volatility

Cryptocurrency market volatility is foremost for investors who intend to incorporate digital currencies in their portfolios (Gkillas et al. 2022). To seize the uneven influence triggered by adverse and optimistic news on the variance of Bitcoin, we consider the exponential generalized autoregressive conditional heteroscedasticity (EGARCH) model suggested by Nelson (1991). The selection of the EGARCH (1,1) model is based on the findings of Naimy and Hayek (2018), who showed the superiority of this specification over the symmetric GARCH (1,1) and exponentially weighted moving average. According to Haroon and Rizvi (2020), the EGARCH model outperforms other specifications due to its ability to permit more stable routine optimization and the lack of parameter restrictions. Furthermore, Güler (2021) found that the EGARCH (1,1) model is the most suitable. The volatility dynamics of the EGARCH (1,1) model are depicted below:

1 $$ln(\sigma_{t}^{2} ) = \, \omega \, + \, \beta ln(\sigma_{t - 1}^{2} ) + \, \gamma \frac{{\varepsilon_{t - 1} }}{{\sqrt {\sigma_{t - 1}^{2} } }} + \, \alpha \left[ {\frac{{\left| {\varepsilon_{t - 1} } \right|}}{{\sqrt {\sigma_{t - 1}^{2} } }} - \sqrt {\frac{2}{\pi }} } \right]$$

where *β* signifies the persistence parameter, and *α* and *γ* describe the size and the sign (leverage) effect, respectively. If *γ* = *0*, the model is entirely symmetric. If *γ* < *0,* adverse shocks boost the instability more than positive shocks. For instance, Chen and Hafner (2019) confirmed that the volatility of Cryptocurrency IndeX (CRIX) soars as the StockTwits sentiment falls. According to Bashir and Kumar (2022), the volatility of the selected cryptocurrencies soars with increased investor focus and unease brought on by pandemic panic. If *γ* > *0,* positive shocks raise the unpredictability more than adverse shocks (Güler 2021; Tiwari et al. 2019). Equation (1) presumes that error terms are normally distributed with a mean equal to $\sqrt{\frac{2}{\pi }}$ (Naimy and Hayek 2018).

#### The nonlinear ARDL bounds testing approach for cointegration

Because market participants obtain information at different moments or interpret situations and facts diversely (Ante 2020), the asymmetric effect must be investigated to gain a better understanding of Bitcoin throughout the pandemic; this approach follows Iqbal et al. (2021), Apergis (2021), and Gaies et al. (2021). As such, Bourghelle et al. (2022) asserted that Bitcoin variability and sentiments may interfere with some asymmetry, complexity, and irregularity. Cheikh et al. (2020) also claimed that substantial price shifts, such as the December 2013 market crash and the late 2017 price levels, highlight the need to investigate whether asymmetric behavior occurs. For instance, Tiwari et al. (2019) demonstrated that digital currencies’ volatilities react more to adverse shocks than positive ones. Fasanya et al. (2022) claimed that nonlinearity is essential for evaluating how investor sentiment influences the interplay between the markets for precious metals and cryptocurrencies. Long et al. (2021) found that when uncertainty lowers, the increase in Bitcoin price outweighs the decline when uncertainty increases. Yarovaya et al. (2021) found asymmetry in herding on bullish and bearish market days, implying panic-forced herding on days when the cryptocurrency market’s value plummeted dramatically. Dias et al. (2022) reported that the consistency of investor sentiment fluctuates across market quantiles, indicating a nonlinear association. Consequently, in line with Iqbal et al. (2021), the variations in the regular number of recently reported COVID-19 instances and fatalities worldwide may influence the returns of Bitcoin differently. Choi and Shin (2022) argued that a shock mainly justifies the increase in Bitcoin prices to its price; however, other shocks, such as the VIX and projected inflation, generally support the drop.

When exploring Bitcoin, a model that captures the potential nonlinearity should be considered (Gajardo et al. 2018). Earlier literature used various techniques, such as regression analysis (Guégan and Renault 2021; Naeem et al. 2020, 2021b; Lyócsa et al. 2020; Sabah 2020; Béjaoui et al. 2021), causality investigation (Guégan and Renault 2021; Güler 2021; Naeem et al. 2020, 2021b; Wu et al. 2021b; Sabah 2020; Béjaoui et al. 2021; Aharon et al. 2020), and VAR/VECM (Güler 2021; Chen et al. 2020; Figà-Talamanca and Patacca 2020; Rognone et al. 2020; Mai et al. 2018; Ciaian et al. 2016; Béjaoui et al. 2021; Zhu et al. 2021). Even if some previous studies estimated the long- and short-run relations (Béjaoui et al. 2021; Ciaian et al. 2016), the asymmetric associations were not assumed. To this end, we apply the NARDL model as in prior studies (Gaies et al. 2021; Rajput et al. 2020; González et al. 2021; Benlagha and Hemrit, 2022). The NARDL model is an asymmetric extension of the ARDL approach. The conventional unrestricted error correction model in the linear ARDL model proposed by Pesaran et al. (2001) is presented as follows:

2 $$\Delta y_{t} = \mu \, + \rho y_{t - 1} + \theta x_{t - 1} + \mathop \sum \limits_{j = 1}^{p - 1} \alpha_{j} \Delta y_{t - j} + \mathop \sum \limits_{j = 0}^{q - 1} \pi_{j} \Delta x_{t - j} + \varepsilon_{t}$$

where *Δ* is the first difference operator, $y_{t}$ is the dependent variable, *μ* signifies the intercept, and $x_{t}$ is a *k* × *1* vector of regressors. $\rho$ and $\theta$ correspond to the long-run coefficients, $\alpha_{j}$ and $\pi_{j}$ denote the short-run coefficients, *p* and *q* depict the lag orders for the dependent and explanatory variables, and $\varepsilon_{t}$ is the error term.

Following Shin et al. (2014), the nonlinear cointegration regression is described below:

3 $$y_{t} = \beta^{ + } x_{t}^{ + } + \beta^{ - } x_{t}^{ - } + u_{t}$$

where $u_{t}$ is a stationary zero-mean error process that indicates deviations from the long-run equilibrium, and ${\beta }^{+}$ and ${\beta }^{-}$ denote the asymmetric long-run parameters. ${x}_{t}$ is the vector of regressors decomposed as follows:

4 $$x_{t} = x_{0} + x_{t}^{ + } + x_{t}^{ - }$$

where $x_{0}$ is a random preliminary value. $x_{t}^{ + }$ and $x_{t}^{ - }$ depict partial sums of positive and negative changes in $x_{t}$ as follows:

5 $$x_{t}^{ + } = \mathop \sum \limits_{j = 1}^{t} \Delta x_{j}^{ + } = \mathop \sum \limits_{j = 1}^{t} max(\Delta x_{j} , 0)$$

6 $$x_{t}^{ - } = \mathop \sum \limits_{j = 1}^{t} \Delta x_{j}^{ - } = \mathop \sum \limits_{j = 1}^{t} min(\Delta x_{j} , 0)$$

By associating Eq. (3) with the linear *ARDL(p,q)* model in Eq. (2), the asymmetric error correction model can be specified as:

7 $$\begin{gathered} \Delta y_{t} = \, \mu \, + \rho y_{t - 1} + \theta^{ + } x_{t - 1}^{ + } + \theta^{ - } x_{t - 1}^{ - } + \mathop \sum \limits_{j = 1}^{p - 1} \alpha_{j} \Delta y_{t - j} \hfill \\ \quad \quad \quad \quad + \mathop \sum \limits_{j = 0}^{q - 1} (\pi_{j}^{ + } \Delta x_{t - j}^{ + } + \pi_{j}^{ - } \Delta x_{t - j}^{ - } ) + \varepsilon_{t} \hfill \\ \end{gathered}$$

where $\theta^{ + }$ = −$\rho \beta^{ + }$ and $\theta^{ - }$ = −$\rho \beta^{ - }$, while $\pi_{j}^{ + }$ and $\pi_{j}^{ - }$ seize the positive and negative short-run adjustments in the explanatory variable $x_{t}$.

Furthermore, several phases should be completed before estimating the NARDL model in Eq. (7). The first step is to ascertain through unit root tests that the included variables are not *I (2)*. Second, the error correction model in Eq. (7) is estimated by traditional ordinary least squares. Third, the bounds test is performed to explore the asymmetric long-run connection among the levels of the series${y}_{t}$, ${x}_{t}^{+}$ and${x}_{t}^{-}$, by applying the F statistic suggested by Pesaran et al. (2001)*.* The null hypothesis of no cointegration *(*$\rho$= ${\theta }^{+}$= ${\theta }^{-}$  = *0)* is assessed versus the alternative of cointegration *(*$\rho$*≠ *$\theta^{ + }$ ≠ $\theta^{ - }$ ≠ *0)*. The fourth step consists of exploring the long-run *(*$\theta^{ + }$ = $\theta^{ - }$*)* and short-run *(*$\pi^{ + }$ = $\pi^{ - }$*)* asymmetries by means of the Wald test. Fifth, the asymmetric cumulative dynamic multiplier effect of a unit change in $x_{t}^{ + }$ and $x_{t}^{ - }$ on $y_{t}$ can be obtained as follows:

8 $$m_{h}^{ + } = \mathop \sum \limits_{j = 0}^{h} \frac{{\partial y_{t + j} }}{{\partial x_{t}^{ + } }}\;{\text{and}}\;m_{h}^{ - } = \mathop \sum \limits_{j = 0}^{h} \frac{{\partial y_{t + j} }}{{\partial x_{t}^{ - } }},\;{\text{h }} = \, 0,{ 1},{ 2}, \, \ldots$$

For Eq. (8), as *h → ∞*, then $m_{h}^{ + }$ → $\beta^{ + }$ and $m_{h}^{ - }$ → $\beta^{ - }$, where the asymmetric long-run coefficients $\beta^{ + }$ = $- \frac{{\theta^{ + } }}{\rho }$ and $\beta^{ - }$ = $- \frac{{\theta^{ - } }}{\rho }$.

The NARDL general model to be estimated in the context of our research takes the following form:

9 $$\begin{gathered} \Delta BTC_{t} = \mu \, + \rho BTC_{t - 1} + \theta_{1}^{ + } COVID\_NEWS_{t - 1}^{ + } + \theta_{1}^{ - } COVID\_NEWS_{t - 1}^{ - } \hfill \\ \quad \quad \quad + \theta_{2}^{ + } COVID\_CASES_{t - 1}^{ + } + \theta_{2}^{ - } COVID\_CASES_{t - 1}^{ - } + \theta_{3}^{ + } VIX_{t - 1}^{ + } + \theta_{3}^{ - } VIX_{t - 1}^{ - } \hfill \\ \quad \quad \quad + \mathop \sum \limits_{i = 1}^{p - 1} \alpha_{i} \Delta BTC_{t - i} + \mathop \sum \limits_{i = 0}^{q} \pi_{1,i}^{ + } \Delta COVID\_NEWS_{t - 1}^{ + } + \mathop \sum \limits_{i = 0}^{q} \pi_{1,i}^{ - } \Delta COVID\_NEWS_{t - 1}^{ - } \hfill \\ \quad \quad \quad + \mathop \sum \limits_{i = 0}^{q} \pi_{2,i}^{ + } \Delta COVID\_CASES_{t - 1}^{ + } + \mathop \sum \limits_{i = 0}^{q} \pi_{2,i}^{ - } \Delta COVID\_CASES_{t - 1}^{ - } \hfill \\ \quad \quad \quad \mathop \sum \limits_{i = 0}^{q} \pi_{3,i}^{ + } \Delta VIX_{t - 1}^{ + } + \mathop \sum \limits_{i = 0}^{q} \pi_{3,i}^{ - } \Delta VIX_{t - 1}^{ - } + \varepsilon_{t} \hfill \\ \end{gathered}$$

where ${BTC}_{t}$ denotes the daily changes of Bitcoin price in period *t*. ${COVID\_NEWS}_{t}$ depict each RavenPack coronavirus-related indices (Panic, Hype, Fake News, Sentiment, Infodemic, and Media Coverage) in period *t*, and ${COVID\_CASES}_{t}$ signifies the daily number of newly reported COVID-19 cases and deaths worldwide in period *t*. ${VIX}_{t}$ indicates the daily change of the Chicago Board Options Exchange volatility index in period *t*, and ${\varepsilon }_{t}$ refers to the error term. Additionally, ${COVID\_NEWS}^{+}$, ${COVID\_NEWS}^{-}$, ${COVID\_CASES}^{+}$, ${COVID\_CASES}^{-}$, ${VIX}^{+}$, and ${VIX}^{-}$ denote the partial sums of positive and negative fluctuations in the explanatory variables.

#### Causality analysis in the frequency domain

The magnitude and direction of causality vary among frequency bands (Granger and Lin 1995), but most conventional approaches to Granger causality disregard the probability that the association’s intensity and path differ over various frequencies (Lemmens et al. 2008). We employ the frequency domain causality test developed by Breitung and Candelon (2006) to examine the causal connection between Bitcoin returns and RavenPack coronavirus-related indices. The effectiveness of using this method ensues from its usage across all periodicities.

Following Geweke (1982), a bivariate vector of time series is considered, ${z}_{t}$ = ${\left[{x}_{t}, {y}_{t}\right]}^{^{\prime}}$, observed at time *t* = *1, …., T*, with a finite-order vector autoregression representation, such as:

10 $$\Theta \left( L \right)z_{t} = \varepsilon_{t}$$

where $\Theta (L)$ = *I–*${\Theta }_{1}L$*—…-* ${\Theta }_{p}{L}^{p}$ is a 2 × 2 lag polynomial with ${L}^{k}{z}_{t}$ = ${z}_{t-k}$. The residual ${\varepsilon }_{t}$ is white noise with $E({\varepsilon }_{t})$ = *0* and $E({\varepsilon }_{t}{\varepsilon }_{t}^{^{\prime}})$ = *Σ*. Since *Σ* is positive definite and symmetric, the Cholesky decomposition ${G}^{^{\prime}}G$ = ${\Sigma }^{-1}$ occurs, where *G* is the inferior triangular matrix and ${G}^{^{\prime}}$ is the upper triangular matrix, such that *E(*${\eta }_{t}{\eta }_{t}^{^{\prime}}$*)* = *I* and ${\eta }_{t}$ = *G* ${\varepsilon }_{t}$. If the system (9) should be stationary, its moving average *(MA)* description is as follows:

11 $$z_{t} = \phi \left( L \right)\varepsilon_{t} = \left[ {\begin{array}{*{20}c} {\Phi_{11} \left( L \right)} & {\Phi_{12} \left( L \right)} \\ {\Phi_{21} \left( L \right)} & {\Phi_{22} \left( L \right)} \\ \end{array} } \right]\left[ {\begin{array}{*{20}c} {\varepsilon_{1t} } \\ {\varepsilon_{2t} } \\ \end{array} } \right]$$

12 $$z_{t} = \Psi \left( L \right)\eta_{t} = \left[ {\begin{array}{*{20}c} {\Psi_{11} \left( L \right)} & {\Psi_{12} \left( L \right)} \\ {\Psi_{21} \left( L \right)} & {\Psi_{22} \left( L \right)} \\ \end{array} } \right]\left[ {\begin{array}{*{20}c} {\eta_{1t} } \\ {\eta_{2t} } \\ \end{array} } \right]$$

where $\Phi (L)$ = *Θ* $({L)}^{-1}$ and $\Psi (L)$ = $\Phi (L){G}^{-1}$. Using this representation, the spectral density of ${x}_{t}$ can be expressed as below:

13 $$f_{x} (\omega ) \, = \frac{1}{2\pi }\left\{ {\left| {\Psi_{11} \left( {e^{ - i\omega } } \right)} \right|^{2} } \right. + \left. {\left| {\Psi_{12} \left( {e^{ - i\omega } } \right)} \right|^{2} } \right\}$$

Furthermore, Geweke (1982) and Hosoya (1991) suggested the following measure of causality:

14 $$M_{y \to x} \left( \omega \right) = {\text{ log}}\left[ {\frac{{2\pi f_{x} \left( \omega \right)}}{{\left| {\Psi_{11} \left( {e^{ - i\omega } } \right)} \right|^{2} }}} \right] = \log \left[ {1 + \frac{{\left| {\Psi_{12} \left( {e^{ - i\omega } } \right)} \right|^{2} }}{{\left| {\Psi_{11} \left( {e^{ - i\omega } } \right)} \right|^{2} }}} \right]$$

If $\left| {\Psi_{12} \left( {e^{ - i\omega } } \right)} \right|^{2}$ = *0*, then $M_{y \to x} (\omega$*)* = *0*, implying that *y* does not Granger cause *x* at periodicity *ω*; hence, the formulation of $\left| {\Psi_{12} \left( {e^{ - i\omega } } \right)} \right|^{2}$ = *0*, can be rendered a state for the absence of Granger causality at frequency *ω*.

If the elements of ${z}_{t}$ are *I(1)* and cointegrated, then, in the frequency domain, the measure of causality can be specified by using the orthogonalized MA description as follows:

15 $$\Delta z_{t} = \left( L \right)\varepsilon_{t} = \tilde{\psi }\left( L \right)\eta_{t}$$

where $\tilde{\psi }$*(L)* = $\tilde{\Phi }$*(L)*$G^{ - 1}$*,* $\eta_{t}$ = *G* $\varepsilon_{t}$, and *G* is a lower triangular matrix, such that *E(*${\eta }_{t}{\eta }_{t}^{^{\prime}})$ = *I*. According to Engle and Granger (1987), in a bivariate cointegrated system, ${\beta }^{^{\prime}}\widetilde{\psi }$*(1)* = *0*, *β* is a cointegration vector, while ${\beta }^{^{\prime}}{z}_{t}$ is stationary. As in the stationary situation, the subsequent causality measure is exhibited as follows:

16 $$M_{y \to x} \left( \omega \right) = \log \left[ {1 + \frac{{\left| {\tilde{\psi }_{12} \left( {e^{ - i\omega } } \right)} \right|^{2} }}{{\left| {\tilde{\psi }_{11} \left( {e^{ - i\omega } } \right)} \right|^{2} }}} \right]$$

The null hypothesis of *y* does not Granger cause *x* is formulated as follows:

17 $$H_{0} :\;M_{y \to x} \left( \omega \right) = 0$$

Breitung and Candelon (2006) exhibited this test by reshaping the association between *x* and *y* in a VAR equation as follows:

18 $$x_{t} = \alpha_{1} x_{t - 1} + \ldots + \alpha_{p} x_{t - p} + \beta_{1} y_{t - 1} + \ldots \beta_{p} y_{t - p} + \varepsilon_{1t}$$

The null hypothesis by Geweke (1982), $M_{y \to x} \left( \omega \right) = 0$, equates to the following null hypothesis:

19 $$H_{0} :\;R\left( \omega \right)\beta = 0$$

where *β* is the vector of the coefficients of *y* and

20 $$R\left( \omega \right) = \left[ {\begin{array}{*{20}c} {cos\left( \omega \right)} & {cos\left( {2\omega } \right)} & \ldots & {cos\left( {p\omega } \right)} \\ {sin\left( \omega \right)} & {sin\left( {2\omega } \right)} & \ldots & {sin\left( {p\omega } \right)} \\ \end{array} } \right]$$

This null hypothesis $\forall \omega \in (0, \pi )$ is tested by an ordinary F statistic distributed as *F(2, T–2p)*, where *2* is the number of restrictions, *p* is the lag length of the VAR model, and *T* is the number of observations.

## Econometric findings

### Summary statistics and correlations

Table 5 presents the basic statistics for all the time series. During the sample period, results show that the highest mean value is registered by Coronavirus Media Coverage Index, whereas Coronavirus Sentiment Index observes the lowest. The mean and median Bitcoin returns are positive, respectively, at 0.5396 and 0.3400%. In line with Wu et al. (2021a), the standard deviation of Bitcoin returns is 4.6169%, suggesting notably high volatility. Furthermore, Aste (2019) documented that prices and sentiment statistics are noisy with substantial volatility. The largest price decline is − 38.18%, and the greatest price rise is 19.56%. The skewness and kurtosis further display the asymmetric and highly leptokurtic distribution of returns. The Jarque−Bera test rejects the normality for all data series following Karaömer (2022). The non-normality of crypto market returns distributions exhibits the rejection of the efficient market assumption, even in its weak form, consistent with Nair (2021).

**Table 5:** Descriptive statistics (raw data)

Variables	Mean	Median	Min	Max	Std. Dev	Skewness	Kurtosis	Jarque–Bera	Prob	Obs
BTC	0.005396	0.0034	−0.3818	0.1956	0.046169	−1.142103	15.58037	2786.027	0.00000	409
PI	2.624377	2.42	0.63	9.21	1.241231	1.803983	7.900164	631.0364	0.00000	409
HI	31.93425	31.89	4.27	69.27	11.69897	0.564388	3.587562	27.59666	0.00000	409
FNI	0.637751	0.56	0.05	2.24	0.32729	1.141626	4.585232	131.6673	0.00000	409
SI	−16.44905	−11.67	−69.92	12.96	20.72573	−0.818649	2.731943	46.90882	0.00000	409
II	48.42301	49.38	9.79	67.67	10.66209	−1.033451	4.63699	118.4705	0.00000	409
MCI	69.6544	72.69	21.91	82.61	9.673174	−2.338832	9.101112	1007.232	0.00000	409
CNC	387,481.9	392,257	98	905,932	255,110.8	0.086215	1.869995	22.26737	0.00002	409
CND	8227.416	7860	1	17,977	4307.619	−0.040538	2.488309	4.574005	0.10157	409
VIX	0.004694	−0.012	−0.2337	0.6164	0.096205	2.099	11.41718	1507.713	0.00000	409

*Source* Authors’ own computations. Notes: Variables’ description is provided in Table 4

**Fig. 1:**
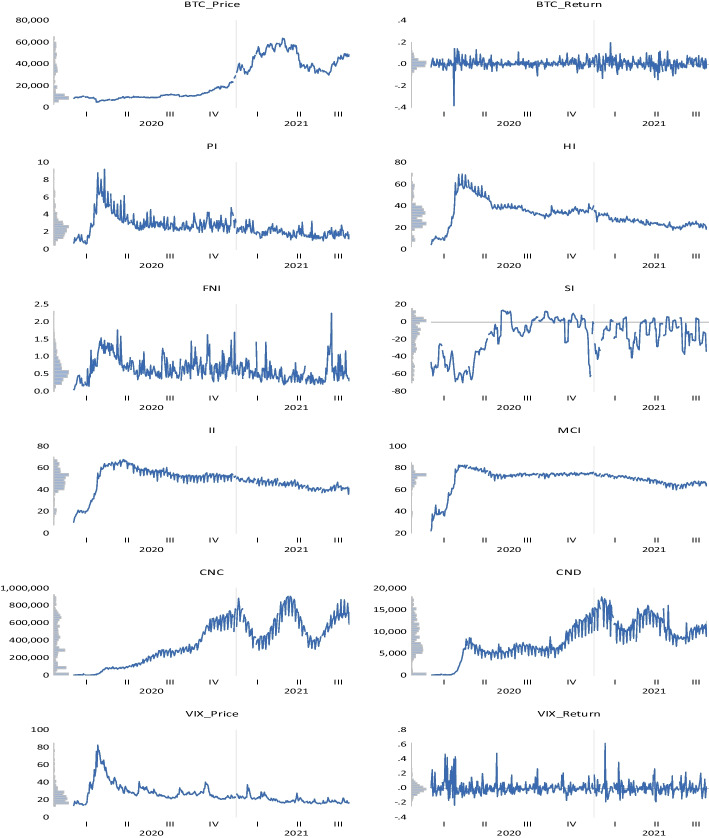
Variable trends from the sample period. *Source* Authors’ own work. Notes: Variables’ description is provided in Table 4

Figure 1 shows the daily evolution of the selected variables. The largest drop of 38.18% in Bitcoin returns was registered on March 12, 2020, while the VIX’s largest decline of 23.37% occurred on March 13, 2020. In the same vein, Akhtaruzzaman et al. (2022) reported that systemic risk soared significantly during the same period but fell to its lowest the subsequent day, suggesting that the advancement of systemic risk-sharing among virtual currencies adjusted rapidly. Mzoughi et al. (2022) ascertained that all markets displayed a substantial persistence in their volatility process, denoting the effects of the crisis. Furthermore, the dynamics of daily Bitcoin returns illustrate evidence of volatility clustering (Bouri et al. 2016; Wang, 2021; Yan et al. 2022; Karaömer 2022); the highest value of Panic Index (PI) (9.21) was registered on March 30, 2021.

Appendix 1 presents the correlations among the selected measures. We notice positive correlations among Bitcoin and all the coronavirus-associated indices except the Sentiment Index. This finding is in line with the assumption of Naeem et al. (2021b) that positive sentiment (optimism) is linked with high returns, although negative sentiment or pessimism drives diminished cryptocurrency returns. Nevertheless, Appendix 2 exhibits the rolling correlations among RavenPack coronavirus-related indices and COVID-19 figures. Like Buigut and Kapar (2021), the correlation coefficients fluctuate extensively, varying from positive to negative. It can be argued that as pandemic cases changed over time, investors gained more knowledge about the disease, identified strategies to accommodate, and the mainstream press became insensitive. This finding is also in line with Bourghelle et al. (2022), who claimed that the market response to the strength of emotion changes over time. Additionally, Akyildirim et al. (2020) reinforced that the conditional correlations of digital currencies and financial market anxiety exhibit time-changing positive interlinkages.

### Asymmetric volatility examination

**Fig. 2:**
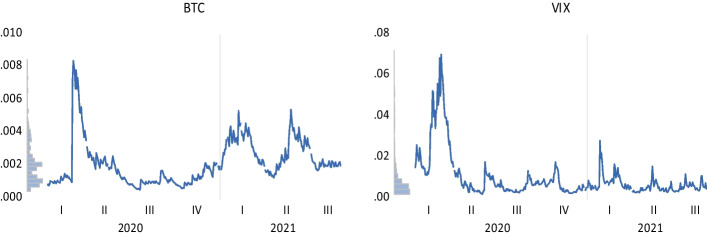
Conditional variance for EGARCH(1,1). *Source* Authors’ own work. Notes: Variables’ description is provided in Table 4

Consistent with Güler (2021), Figà–Talamanca and Patacca (2020), López–Cabarcos et al. (2021), Bouri et al. (2016), Wang (2021), Kakinaka and Umeno (2021b), and Cheikh et al. (2020), we estimate an EGARCH (1,1) model. Appendices 3 and 4 present the estimation outcomes for Bitcoin and VIX. Accordingly, since *C(4)* in Appendix 3 indicates that the leverage parameter is not statistically significant, we conclude that no asymmetric effect occurs for Bitcoin; hence, volatility does not rise more in reaction to positive shocks than in response to adverse shocks. The outcomes are in line with Wang (2021) and Nair (2021) but contrary to Apergis (2021), Iqbal et al. (2021), Baur and Dimpfl (2018), Güler (2021), and Bashir and Kumar (2022). Furthermore, Kakinaka and Umeno (2021b) and Cheikh et al. (2020) concluded that the asymmetric effect could not be statistically confirmed. According to Nair (2021), the profit (deficit) registered in the preceding period is quite significant in generating shortfalls (rewards) to dealers through the following day in both high- and low-price extreme shifts of crypto markets. Overall, the FOMO behavior identified in prior studies (Güler 2021; Baur and Dimpfl 2018) is not supported, suggesting that prudence rather than feelings drive the Bitcoin market; however, the empirical outcomes from Appendix 4 support that in the case of VIX the leverage effect is positive and statistically significant. This finding suggests that positive shocks can significantly impact volatility more than adverse ones. Cheikh et al. (2020) argued that investors seeking a hedge against a depressed stock market would transfer volatility and uncertainty to cryptocurrency markets throughout market tumult. Additionally, Fig. 2 exhibits that the conditional variance of VIX is greater than that of Bitcoin.

### Stationarity investigation

Appendix 5 reveals the outcomes of stationarity tests performed with the traditional methods. The NARDL model is estimated regardless of whether the variables are integrated of order 0 or 1 (*I(0) or I(1)*). Nevertheless, the NARDL framework cannot be considered if one of the variables is *I(2)* since the value of the F-test related to the bounds testing cointegration approach is invalid.

We examine the order of integration between the variables through the ADF test proposed by Dickey and Fuller (1979) and the PP test suggested by Phillips and Perron (1988), following the extant literature (Polat et al. 2022; Mokni et al. 2022; Burggraf et al. 2021; Ghosh 2020; Sahoo 2021; Sahoo and Rath 2022). Furthermore, because the ADF and PP tests are supposed to be biased toward *I(1)* inferences, we employ the KPSS test of Kwiatkowski et al. (1992), in line with Kakinaka and Umeno (2021b), Sahoo (2021), and Karaömer (2022). Both ADF and PP tests rely on the null hypothesis that the variables comprise a unit root (follows a random walk) and therefore are not stationary, against the alternative hypothesis that a stationary process generated the data series; however, the KPSS test sets out as a null hypothesis that the variables are stationary. The results reveal that the variables are either *I(0)* or *I(1)*, but none of the measures is stationary at the second difference, thus supporting the appropriateness of the NARDL model.

Furthermore, the traditional stationarity tests may misleadingly establish that the variables are *I(1)* or *I(2)* when breaks occur in the series; therefore, we employ the test recommended by Zivot and Andrews (1992) to prove the rejection of *I(2)* measures. Appendix 6 provides the results of the ZA test. The outcomes reinforce that none of the variables is *I(2)*, highlighting time breaks in the data, in line with Burggraf et al. (2021), Ghosh (2020), and Iqbal et al. (2021). Essentially, the identified breaks associate with the COVID-19 waves.

### Checking for nonlinear dependence

To explore the likelihood of nonlinear dependence among Bitcoin returns and RavenPack coronavirus-related indices, we performed the Brock–Dechert–Scheinkman (BDS) test suggested by Broock et al. (1996). The BDS test is a nonparametric check robust to the structure of nonlinearity in the data. Table 6 shows the outcomes of the BDS test.

**Table 6:** Nonlinearity Brock-Dechert-Scheinkman (BDS) test

Variables	Embedding Dimension = m				
	m = 2	m = 3	m = 4	m = 5	m = 6
BTC	0.007296	0.014082*	0.017278**	0.023407***	0.027509***
PI	0.108314***	0.178543***	0.220047***	0.240719***	0.257101***
HI	0.174347***	0.295623***	0.376935***	0.432012***	0.472148***
FNI	0.07069***	0.11507***	0.136611***	0.144885***	0.143907***
SI	0.168912***	0.280105***	0.349911***	0.393345***	0.417379***
II	0.163368***	0.281909***	0.360839***	0.415235***	0.45656***
MCI	0.164117***	0.27832***	0.355746***	0.411665***	0.451416***
CNC	0.163014***	0.283858***	0.370032***	0.429234***	0.472059***
CND	0.131594***	0.238702***	0.311789***	0.359086***	0.393218***
VIX	0.024614***	0.052417***	0.069922***	0.075609***	0.075304***

*Source* Authors’ own computations. Notes: Superscripts *, **, ***represent the significance at 10%, 5%, and 1% levels, respectively. Variables’ description is provided in Table 4

The BDS test rejects the null hypothesis of linearity consistent with Mokni et al. (2022), Raza et al. (2022a, b), namely independent and identically distributed residuals across various embedding dimensions. Therefore, all incorporated variables are nonlinear, proving the chaotic behavior in the time series data. Likewise, the nonlinear modeling approach is appropriate for this study’s objectives.

### Testing for cointegration

Next, we examine the cointegration link among the variables; Table 7 reports the results of the asymmetric cointegration test. The F-statistics are greater than the upper bound values involving the rejection of the null hypothesis of no cointegration; hence, the results show evidence for long-run relationships (cointegration) in all cases.

**Table 7:** Bounds test for nonlinear cointegration

Model no	Model specification	NARDL specification	F-statistic	Critical Value		
1	F (BTC_t_/PI^+^, PI^−^, CNC^+^, CNC^−^, VIX^+^, VIX^−^)	NARDL (1, 0, 0, 3, 3, 0, 0)	82.29521***	1%	I (0)	2.88
2	F (BTC_t_/HI^+^, HI^−^, CNC^+^, CNC^−^, VIX^+^, VIX^−^)	NARDL (1, 0, 0, 3, 3, 0, 0)	80.86306***		I (1)	3.99
3	F (BTC_t_/FNI^+^, FNI^−^, CNC^+^, CNC^−^, VIX^+^, VIX^−^)	NARDL (1, 2, 1, 2, 4, 0, 0)	87.20392***	5%	I (0)	2.27
4	F (BTC_t_/SI^+^, SI^−^, CNC^+^, CNC^−^, VIX^+^, VIX^−^)	NARDL (1, 1, 0, 0, 0, 0, 0)	85.21631***		I (1)	3.28
5	F (BTC_t_/II^+^, II^−^, CNC^+^, CNC^−^, VIX^+^, VIX^−^)	NARDL (1, 0, 1, 3, 3, 0, 0)	80.22583***	10%	I (0)	1.99
6	F (BTC_t_/MCI^+^, MCI^−^, CNC^+^, CNC^−^, VIX^+^, VIX^−^)	NARDL (1, 0, 0, 3, 3, 0, 0)	80.21185***		I (1)	2.94
7	F (BTC_t_/PI^+^, PI^−^, CND^+^, CND^−^, VIX^+^, VIX^−^)	NARDL (1, 0, 0, 0, 0, 0, 0)	87.11501***			
8	F (BTC_t_/HI^+^, HI^−^, CND^+^, CND^−^, VIX^+^, VIX^−^)	NARDL (1, 0, 0, 0, 0, 0, 0)	84.97346***			
9	F (BTC_t_/FNI^+^, FNI^−^, CND^+^, CND^−^, VIX^+^, VIX^−^)	NARDL (1, 2, 1, 0, 0, 0, 0)	89.54702***			
10	F (BTC_t_/SI^+^, SI^−^, CND^+^, CND^−^, VIX^+^, VIX^−^)	NARDL (1, 1, 0, 0, 0, 0, 0)	84.24209***			
11	F (BTC_t_/II^+^, II^−^, CND^+^, CND^−^, VIX^+^, VIX^−^)	NARDL (1, 0, 0, 0, 0, 1, 0)	77.60079***			
12	F (BTC_t_/MCI^+^, MCI^−^, CND^+^, CND^−^, VIX^+^, VIX^−^)	NARDL (1, 0, 0, 0, 0, 0, 0)	83.92854***			

*Source* Authors’ own computations. Notes: Superscripts *, **, *** represent the significance at 10%, 5%, and 1% levels, respectively. Model selection method: Akaike info criterion (AIC). Variables’ description is provided in Table 4

### NARDL outcomes

Table 8 presents the estimates of NARDL models 1–6, covering the daily number of newly reported COVID-19 cases worldwide. Error correction term (ECT) is negative and statistically significant at 1%, thus confirming the ability of the short-run disequilibrium to adjust at long-run equilibrium. The long-term impact coefficients of the increase in the Coronavirus PI, Coronavirus Hype Index (HI), Coronavirus Fake News Index (FNI), and Coronavirus Infodemic Index (II) on daily changes in Bitcoin price are 0.008191 $({\mathrm{L}}_{\mathrm{PI}}^{+}$), 0.000818 $({\mathrm{L}}_{\mathrm{HI}}^{+}$), 0.035054 $({\mathrm{L}}_{\mathrm{FNI}}^{+}$), and 0.001512 $({\mathrm{L}}_{\mathrm{II}}^{+}$), being statistically significant. This result shows that the rise in PI, HI, FNI, and II has a significant augmenting effect on Bitcoin returns; that is, when the PI, HI, FNI, and II rise by 1%, Bitcoin returns rise by 0.008191%, 0.000818%, 0.035054%, and 0.001512%, respectively. Additionally, the long-term impact coefficients of the decline in PI, HI, FNI, and II on Bitcoin returns are 0.007009 $({\mathrm{L}}_{\mathrm{PI}}^{-}$), 0.0007 $({\mathrm{L}}_{\mathrm{HI}}^{-})$, 0.03341 ${(\mathrm{L}}_{\mathrm{FNI}}^{-})$, and 0.001215 ${(\mathrm{L}}_{\mathrm{II}}^{+})$, respectively, indicating that the decrease of PI, HI, FNI, and II significantly promotes Bitcoin’s daily changes. The outcomes are in line with Rognone et al. (2020), suggesting investor fervor for Bitcoin regardless of the sentiment of the news. Furthermore, the positive impact of the pandemic sentiment on Bitcoin returns is consistent with Goodell and Goutte (2021). Additionally, Mahdi and Al–Abdulla (2022) showed that Bitcoin returns increase as the frequency of fear-related headlines increases. Following Shrotryia and Kalra (2021) and Marobhe (2022), the empirical findings reject any uneven behavioral shape throughout the pandemic disorder. The outcomes confirm Akhtaruzzaman et al. (2022), indicating that Bitcoin is systemically reliable and has a reduced potential to trigger structural disturbances. The quantitative outcomes also support Sifat (2021), who advocated dissociating digital currencies from global sentiments; however, the long-term impact coefficient of the decrease in Coronavirus Sentiment Index (SI) shows a negative influence on Bitcoin returns, whereas the long-run impact coefficient of the rise in SI is not statistically significant. With reference to the Coronavirus Media Coverage Index (MCI), the long-run impact coefficient of the increase in MCI is statistically significant, revealing a positive influence on daily changes of Bitcoin price. Additionally, the results of the Wald test show that the long-term asymmetric impact on Bitcoin returns is statistically significant only in the cases of PI and II.

**Table 8:** NARDL long run and short run estimates (daily number of new reported COVID-19 cases worldwide included)

Model 1		Model 2		Model 3		Model 4		Model 5		Model 6	
$\mathrm{ECT}$	−1.090046*** (0.042107)	$\mathrm{ECT}$	−1.095174*** (0.042679)	$\mathrm{ECT}$	−1.132591*** (0.042498)	$\mathrm{ECT}$	−1.11978*** (0.042515)	$\mathrm{ECT}$	−1.094086*** (0.042804)	$\mathrm{ECT}$	−1.096896*** (0.042919)
*Long-run and short-run coefficients*											
Const	0.011766 (0.02905)	Const	0.003942 (0.031316)	Const	−0.00041 (0.027761)	Const	0.01041 (0.022178)	Const	0.016295 (0.0315)	Const	−0.014911 (0.027113)
${\mathrm{BTC}}_{\mathrm{t}-1}$	−1.090046*** (0.046904)	${\mathrm{BTC}}_{\mathrm{t}-1}$	−1.095174*** (0.047337)	${\mathrm{BTC}}_{\mathrm{t}-1}$	−1.132591*** (0.047067)	${\mathrm{BTC}}_{\mathrm{t}-1}$	−1.11978*** (0.046833)	${\mathrm{BTC}}_{\mathrm{t}-1}$	−1.094086*** (0.047676)	${\mathrm{BTC}}_{\mathrm{t}-1}$	−1.096896*** (0.047675)
${\mathrm{PI}}^{+}$	0.008929*** (0.002856)	${\mathrm{HI}}^{+}$	0.000896* (0.000499)	${\mathrm{FNI}}^{+}$	0.039701*** (0.010661)	${\mathrm{SI}}_{\mathrm{t}-1}^{+}$	−0.000149 (0.00018)	${\mathrm{II}}^{+}$	0.001655* (0.000848)	${\mathrm{MCI}}^{+}$	0.001342* (0.000722)
${\mathrm{PI}}^{-}$	0.00764*** (0.002485)	${\mathrm{HI}}^{-}$	0.000766** (0.000329)	${\mathrm{FNI}}_{\mathrm{t}-1}^{-}$	0.03784*** (0.010433)	${\mathrm{SI}}^{-}$	−0.00022* (0.00013)	${\mathrm{II}}_{\mathrm{t}-1}^{-}$	0.001329* (0.000688)	${\mathrm{MCI}}^{-}$	0.000859 (0.00057)
${\mathrm{CNC}}_{\mathrm{t}-1}^{+}$	−0.004168 (0.004575)	${\mathrm{CNC}}_{\mathrm{t}-1}^{+}$	−0.005491 (0.007048)	${\mathrm{CNC}}_{\mathrm{t}-1}^{+}$	−0.000792 (0.003991)	${\mathrm{CNC}}^{+}$	0.003035 (0.004543)	${\mathrm{CNC}}_{\mathrm{t}-1}^{+}$	−0.010901 (0.008179)	${\mathrm{CNC}}_{\mathrm{t}-1}^{+}$	−0.006703 (0.006833)
${\mathrm{CNC}}_{\mathrm{t}-1}^{-}$	−0.000471 (0.003542)	${\mathrm{CNC}}_{\mathrm{t}-1}^{-}$	−0.001544 (0.006103)	${\mathrm{CNC}}_{\mathrm{t}-1}^{-}$	0.004079 (0.00284)	${\mathrm{CNC}}^{-}$	0.007222^*^ (0.004311)	${\mathrm{CNC}}_{\mathrm{t}-1}^{-}$	−0.007015 (0.006038)	${\mathrm{CNC}}_{\mathrm{t}-1}^{-}$	−0.003014 (0.004615)
${\mathrm{VIX}}^{+}$	−0.164421^***^ (0.02411)	${\mathrm{VIX}}^{+}$	−0.157511^***^ (0.024488)	${\mathrm{VIX}}^{+}$	−0.161608^***^ (0.023664)	${\mathrm{VIX}}^{+}$	−0.1567^***^ (0.023642)	${\mathrm{VIX}}^{+}$	−0.164333^***^ (0.024033)	${\mathrm{VIX}}^{+}$	−0.16386^***^ (0.024246)
${\mathrm{VIX}}^{-}$	−0.163397*** (0.022702)	${\mathrm{VIX}}^{-}$	−0.162211*** (0.022835)	${\mathrm{VIX}}^{-}$	−0.163935*** (0.022708)	${\mathrm{VIX}}^{-}$	−0.158306*** (0.022925)	${\mathrm{VIX}}^{-}$	−0.164061*** (0.022973)	${\mathrm{VIX}}^{-}$	−0.163882*** (0.022868)
$\Delta {\mathrm{CNC}}^{+}$	0.028717** (0.014535)	$\Delta {\mathrm{CNC}}^{+}$	0.032861** (0.014497)	$\Delta {\mathrm{FNI}}^{+}$	0.025312** (0.012096)	$\Delta {\mathrm{SI}}^{+}$	0.000807* (0.000483)	$\Delta {\mathrm{II}}^{-}$	−0.00105 (0.00129)	$\Delta {\mathrm{CNC}}^{+}$	0.031113** (0.014683)
$\Delta {\mathrm{CNC}}_{\mathrm{t}-1}^{+}$	−0.023874* (0.013517)	$\Delta {\mathrm{CNC}}_{\mathrm{t}-1}^{+}$	−0.021712 (0.014058)	$\Delta {\mathrm{FNI}}_{\mathrm{t}-1}^{+}$	−0.037215** (0.016586)			$\Delta {\mathrm{CNC}}^{+}$	0.029177** (0.014815)	$\Delta {\mathrm{CNC}}_{\mathrm{t}-1}^{+}$	−0.021358 (0.013731)
$\Delta {\mathrm{CNC}}_{\mathrm{t}-2}^{+}$	0.025533** (0.011109)	$\Delta {\mathrm{CNC}}_{\mathrm{t}-2}^{+}$	0.028266** (0.011359)	$\Delta {\mathrm{FNI}}^{-}$	−0.016338 (0.016756)			$\Delta {\mathrm{CNC}}_{\mathrm{t}-1}^{+}$	−0.016774 (0.014142)	$\Delta {\mathrm{CNC}}_{\mathrm{t}-2}^{+}$	0.027311** (0.011297)
$\Delta {\mathrm{CNC}}^{-}$	−0.001122 (0.014702)	$\Delta {\mathrm{CNC}}^{-}$	−0.008519 (0.014833)	$\Delta {\mathrm{CNC}}^{+}$	0.028241** (0.014226)			$\Delta {\mathrm{CNC}}_{\mathrm{t}-2}^{+}$	0.028706** (0.011464)	$\Delta {\mathrm{CNC}}^{-}$	−0.012482 (0.015553)
$\Delta {\mathrm{CNC}}_{\mathrm{t}-1}^{-}$	0.039652** (0.018272)	$\Delta {\mathrm{CNC}}_{\mathrm{t}-1}^{-}$	0.043047** (0.018396)	$\Delta {\mathrm{CNC}}_{\mathrm{t}-1}^{+}$	−0.019469 (0.013647)			$\Delta {\mathrm{CNC}}^{-}$	−0.002625 (0.016823)	$\Delta {\mathrm{CNC}}_{\mathrm{t}-1}^{-}$	0.040951** (0.018305)
$\Delta {\mathrm{CNC}}_{\mathrm{t}-2}^{-}$	−0.040723** (0.017395)	$\Delta {\mathrm{CNC}}_{\mathrm{t}-2}^{-}$	−0.041628** (0.017463)	$\Delta {\mathrm{CNC}}^{-}$	−0.011874 (0.015298)			$\Delta {\mathrm{CNC}}_{\mathrm{t}-1}^{-}$	0.041516** (0.018362)	$\Delta {\mathrm{CNC}}_{\mathrm{t}-2}^{-}$	−0.040451** (0.017309)
				$\Delta {\mathrm{CNC}}_{\mathrm{t}-1}^{-}$	0.03323* (0.017541)			$\Delta {\mathrm{CNC}}_{\mathrm{t}-2}^{-}$	−0.036842** (0.017236)		
				$\Delta {\mathrm{CNC}}_{\mathrm{t}-2}^{-}$	−0.026595 (0.016372)						
				$\Delta {\mathrm{CNC}}_{\mathrm{t}-3}^{-}$	−0.032454** (0.013145)						
*Long-run asymmetric effects*											
${\mathrm{L}}_{\mathrm{PI}}^{+}$	0.008191*** (0.002634)	${\mathrm{L}}_{\mathrm{HI}}^{+}$	0.000818* (0.000455)	${\mathrm{L}}_{\mathrm{FNI}}^{+}$	0.035054*** (0.00931)	${\mathrm{L}}_{\mathrm{SI}}^{+}$	−0.000133 (0.00016)	${\mathrm{L}}_{\mathrm{II}}^{+}$	0.001512* (0.00077)	${\mathrm{L}}_{\mathrm{MCI}}^{+}$	0.001223* (0.000654)
${\mathrm{L}}_{\mathrm{PI}}^{-}$	0.007009*** (0.00229)	${\mathrm{L}}_{\mathrm{HI}}^{-}$	0.0007** (0.000299)	${\mathrm{L}}_{\mathrm{FNI}}^{-}$	0.03341*** (0.009118)	${\mathrm{L}}_{\mathrm{SI}}^{-}$	−0.000196* (0.000116)	${\mathrm{L}}_{\mathrm{II}}^{-}$	0.001215* (0.000625)	${\mathrm{L}}_{\mathrm{MCI}}^{-}$	0.000783 (0.000516)
${\mathrm{L}}_{\mathrm{CNC}}^{+}$	−0.003823 (0.004201)	${\mathrm{L}}_{\mathrm{CNC}}^{+}$	−0.005014 (0.006435)	${\mathrm{L}}_{\mathrm{CNC}}^{+}$	−0.0007 (0.003524)	${\mathrm{L}}_{\mathrm{CNC}}^{+}$	0.002711 (0.004055)	${\mathrm{L}}_{\mathrm{CNC}}^{+}$	−0.009964 (0.007447)	${\mathrm{L}}_{\mathrm{CNC}}^{+}$	−0.006111 (0.006208)
${\mathrm{L}}_{\mathrm{CNC}}^{-}$	−0.000432 (0.00325)	${\mathrm{L}}_{\mathrm{CNC}}^{-}$	−0.00141 (0.005575)	${\mathrm{L}}_{\mathrm{CNC}}^{-}$	0.003602 (0.002496)	${\mathrm{L}}_{\mathrm{CNC}}^{-}$	0.006449* (0.00383)	${\mathrm{L}}_{\mathrm{CNC}}^{-}$	−0.006411 (0.00551)	${\mathrm{L}}_{\mathrm{CNC}}^{-}$	−0.002748 (0.004203)
${\mathrm{L}}_{\mathrm{VIX}}^{+}$	−0.150839*** (0.023894)	${\mathrm{L}}_{\mathrm{VIX}}^{+}$	−0.143823*** (0.024099)	${\mathrm{L}}_{\mathrm{VIX}}^{+}$	−0.142689*** (0.022549)	${\mathrm{L}}_{\mathrm{VIX}}^{+}$	−0.139938*** (0.022723)	${\mathrm{L}}_{\mathrm{VIX}}^{+}$	−0.150201*** (0.023827)	${\mathrm{L}}_{\mathrm{VIX}}^{+}$	−0.149385*** (0.023932)
${\mathrm{L}}_{\mathrm{VIX}}^{-}$	−0.1499*** (0.022607)	${\mathrm{L}}_{\mathrm{VIX}}^{-}$	−0.148114*** (0.022629)	${\mathrm{L}}_{\mathrm{VIX}}^{-}$	−0.144743*** (0.02174)	${\mathrm{L}}_{\mathrm{VIX}}^{-}$	−0.141372*** (0.022083)	${\mathrm{L}}_{\mathrm{VIX}}^{-}$	−0.149953*** (0.022858)	${\mathrm{L}}_{\mathrm{VIX}}^{-}$	−0.149405*** (0.022651)
Const	0.010794 (0.026645)	Const	0.003599 (0.028592)	Const	−0.000362 (0.024511)	Const	0.009296 (0.019797)	Const	0.014894 (0.028755)	Const	−0.013594 (0.024736)
*Long run asymmetry tests – Wald statistics*											
${\mathrm{W}}_{\mathrm{LR}}(\mathrm{PI})$	4.487301**	${\mathrm{W}}_{\mathrm{LR}}(\mathrm{HI})$	0.3338	${\mathrm{W}}_{\mathrm{LR}}(\mathrm{FNI})$	2.139061	${\mathrm{W}}_{\mathrm{LR}}(\mathrm{SI})$	0.148092	${\mathrm{W}}_{\mathrm{LR}}(\mathrm{II})$	4.581732**	${\mathrm{W}}_{\mathrm{LR}}(\mathrm{MCI})$	1.265293
${\mathrm{W}}_{\mathrm{LR}}(\mathrm{CNC})$	2.739795*	${\mathrm{W}}_{\mathrm{LR}}(\mathrm{CNC})$	3.337275*	${\mathrm{W}}_{\mathrm{LR}}(\mathrm{CNC})$	2.451346	${\mathrm{W}}_{\mathrm{LR}}(\mathrm{CNC})$	1.772852	${\mathrm{W}}_{\mathrm{LR}}(\mathrm{CNC})$	3.37373*	${\mathrm{W}}_{\mathrm{LR}}(\mathrm{CNC})$	3.355494*
${\mathrm{W}}_{\mathrm{LR}}(\mathrm{VIX})$	0.001809	${\mathrm{W}}_{\mathrm{LR}}(\mathrm{VIX})$	1.109015	${\mathrm{W}}_{\mathrm{LR}}(\mathrm{VIX})$	0.316811	${\mathrm{W}}_{\mathrm{LR}}(\mathrm{VIX})$	0.188459	${\mathrm{W}}_{\mathrm{LR}}(\mathrm{VIX})$	0.113445	${\mathrm{W}}_{\mathrm{LR}}(\mathrm{VIX})$	0.4769
*Statistics and diagnostics*											
R-sq	0.190674	R−sq	0.18174	R−sq	0.215594	R−sq	0.158729	R−sq	0.179996	R−sq	0.177612
Adj R-sq	0.163765	Adj R−sq	0.154534	Adj R−sq	0.183163	Adj R−sq	0.14182	Adj R−sq	0.15056	Adj R−sq	0.150269
F-stat	7.085999***	F−stat	6.680244***	F−stat	6.647919***	F−stat	9.386746***	F−stat	6.114826***	F−stat	6.495744***
D-W stat	2.060461	D−W stat	2.058759	D−W stat	2.044533	D−W stat	2.03404	D−W stat	2.063541	D−W stat	2.070825
${\upchi }_{\mathrm{SC}}^{2} (1)$	2.366963 [0.1247]	${\upchi }_{\mathrm{SC}}^{2} (1)$	2.467295 [0.117]	${\upchi }_{\mathrm{SC}}^{2} (1)$	1.263353 [0.2617]	${\upchi }_{\mathrm{SC}}^{2} (1)$	1.098021 [0.2953]	${\upchi }_{\mathrm{SC}}^{2} (1)$	2.856843 [0.0918]	${\upchi }_{\mathrm{SC}}^{2} (1)$	3.44255 [0.0643]
${\upchi }_{\mathrm{SC}}^{2} (2)$	1.217663 [0.297]	${\upchi }_{\mathrm{SC}}^{2} (2)$	1.238976 [0.2908]	${\upchi }_{\mathrm{SC}}^{2} (2)$	0.816957 [0.4425]	${\upchi }_{\mathrm{SC}}^{2} (2)$	0.552424 [0.576]	${\upchi }_{\mathrm{SC}}^{2} (2)$	1.441961 [0.2377]	${\upchi }_{\mathrm{SC}}^{2} (2)$	1.756983 [0.1739]
${\upchi }_{\mathrm{HET}}^{2} (1)$	0.118551 [0.7308]	${\upchi }_{\mathrm{HET}}^{2} (1)$	0.050962 [0.8215]	${\upchi }_{\mathrm{HET}}^{2} (1)$	0.090182 [0.7641]	${\upchi }_{\mathrm{HET}}^{2} (1)$	0.003739 [0.9513]	${\upchi }_{\mathrm{HET}}^{2} (1)$	0.016026 [0.8993]	${\upchi }_{\mathrm{HET}}^{2} (1)$	0.047376 [0.8278]
${\upchi }_{\mathrm{HET}}^{2} (2)$	0.062791 [0.9391]	${\upchi }_{\mathrm{HET}}^{2} (2)$	0.031419 [0.9691]	${\upchi }_{\mathrm{HET}}^{2} (2)$	0.045698 [0.9553]	${\upchi }_{\mathrm{HET}}^{2} (2)$	0.004194 [0.9958]	${\upchi }_{\mathrm{HET}}^{2} (2)$	0.008891 [0.9911]	${\upchi }_{\mathrm{HET}}^{2} (2)$	0.02622 [0.9741]
${\upchi }_{\mathrm{NORM}}^{2}$	1196.586 [0.0000]	${\upchi }_{\mathrm{NORM}}^{2}$	1146.037 [0.0000]	${\upchi }_{\mathrm{NORM}}^{2}$	1337.827 [0.0000]	${\upchi }_{\mathrm{NORM}}^{2}$	1381.666 [0.0000]	${\upchi }_{\mathrm{NORM}}^{2}$	1056.104 [0.0000]	${\upchi }_{\mathrm{NORM}}^{2}$	1240.097 [0.0000]
${\upchi }_{\mathrm{RESET}}^{2}$	0.364455 [0.5464]	${\upchi }_{\mathrm{RESET}}^{2}$	0.007184 [0.9325]	${\upchi }_{\mathrm{RESET}}^{2}$	0.174737 [0.6762]	${\upchi }_{\mathrm{RESET}}^{2}$	0.075537 [0.7836]	${\upchi }_{\mathrm{RESET}}^{2}$	0.754172 [0.4512]	${\upchi }_{\mathrm{RESET}}^{2}$	0.106922 [0.9149]

*Source* Authors’ own computations. Notes: Superscripts ^*^, ^**^, ^***^ represent the significance at 10%, 5%, and 1% levels, respectively. The superscript + and – defines positive and negative partial sum. ${\mathrm{L}}^{+}$ and ${\mathrm{L}}^{-}$ are the computed long-run coefficients associated with positive and negative shocks, respectively. ${\mathrm{W}}_{\mathrm{LR}}$ denotes the Wald statistic for the long-run symmetry. ${\mathrm{W}}_{\mathrm{LR}}$ denotes the Wald statistic for the long-run symmetry, which tests the null hypothesis of ${\theta }^{+}$ =${\theta }^{-}$. ${\mathrm{\rm X}}_{\mathrm{SC}}^{2}$ denotes the Breusch-Godfrey Serial Correlation LM Test (first and second lag). ${\mathrm{\rm X}}_{\mathrm{HET}}^{2}$ denotes the Heteroskedasticity Test: ARCH (first and second lag). ${\mathrm{\rm X}}_{\mathrm{RESET}}^{2}$ denotes the Ramsey RESET Test of Misspecification. ${\mathrm{\rm X}}_{\mathrm{NORM}}^{2}$ denotes the Jarque–Bera test. The p-values of diagnostic tests are in []. Variables’ description is provided in Table 4

As for the daily number of newly reported COVID-19 cases worldwide, contrary to Sarkodie et al. (2022), the positive shock ${(\mathrm{L}}_{\mathrm{CNC}}^{+})$ and negative shock ${(\mathrm{L}}_{\mathrm{CNC}}^{-})$, as shown in Table 8, are almost negative but not statistically significant. Furthermore, like Gaies et al. (2021), in the long-run, positive ${(\mathrm{L}}_{\mathrm{VIX}}^{+})$ and negative ${(\mathrm{L}}_{\mathrm{VIX}}^{-})$ shocks to VIX negatively impact Bitcoin returns at the 1% significance level. The outcomes are contrary to Anamika et al. (2021) but consistent with Bouri et al. (2016), who found that Bitcoin volatility inversely associates with US uncertainty, as well as Su et al. (2022).

**Fig. 3:**
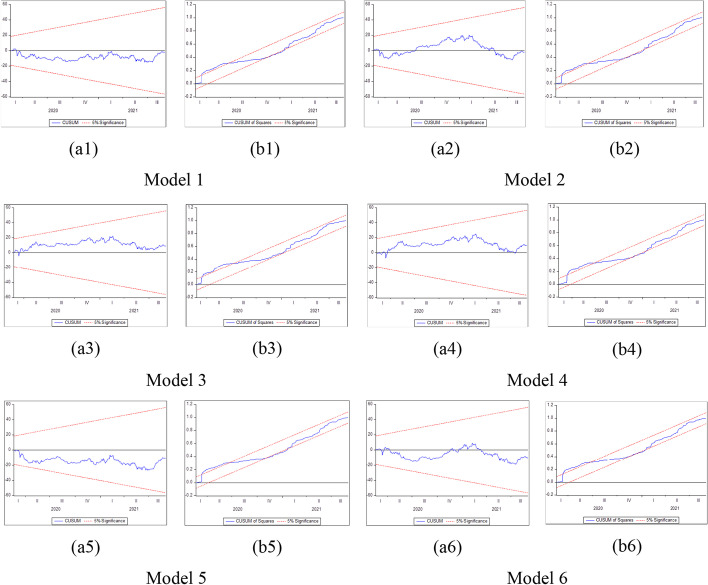
NARDL plots of cumulative sum of recursive residuals—CUSUM (a1–a6) and cumulative sum square of recursive residuals—CUSUMSQ (b1–b6) for Models 1–6. *Source* Authors’ own work. Notes: The blue line is the solid line while the red lines that bounded the blue line are the critical bounds at 0.5. Variables’ description is provided in Table 4

Regarding the results of diagnostic tests of Table 8, Breusch–Godfrey serial correlation LM test (${\upchi }_{\mathrm{SC}}^{2})$ and ARCH heteroskedasticity test $({\upchi }_{\mathrm{HET}}^{2})$ indicate that the null hypothesis (with no serial autocorrelation and heteroskedasticity in the residuals) cannot be rejected. Furthermore, in line with Gaies et al. (2021) and Rajput et al. (2020), the stability of the NARDL models 1–6 is checked and confirmed through the cumulative sum (CUSUM) and the CUSUM of squares (CUSUMQ) tests proposed by Brown et al. (1975); Fig. 3 presents the results. The CUSUM test provides a plot of the long- and short-term coefficients of the cumulative error terms of the number of observations with a 5% confidence interval, while the CUSUMQ test assesses the coefficients by squaring the cumulative error terms (Vurur 2021). The recursive and squared recursive residuals are drawn against breakpoints for CUSUM and CUSUMQ, respectively. If any point outstrips the 5% level of significance symbolized by the straight (red) lines, the null assumption that the parameters are stable is rejected (Gaies et al. 2021).

Generally, the blue lines do not outstrip the two red lines, suggesting that structural stability is supported for both short- and long-term estimates; hence, no significant structural variations compromise the stability of the estimates of the NARDL models.

**Fig. 4:**
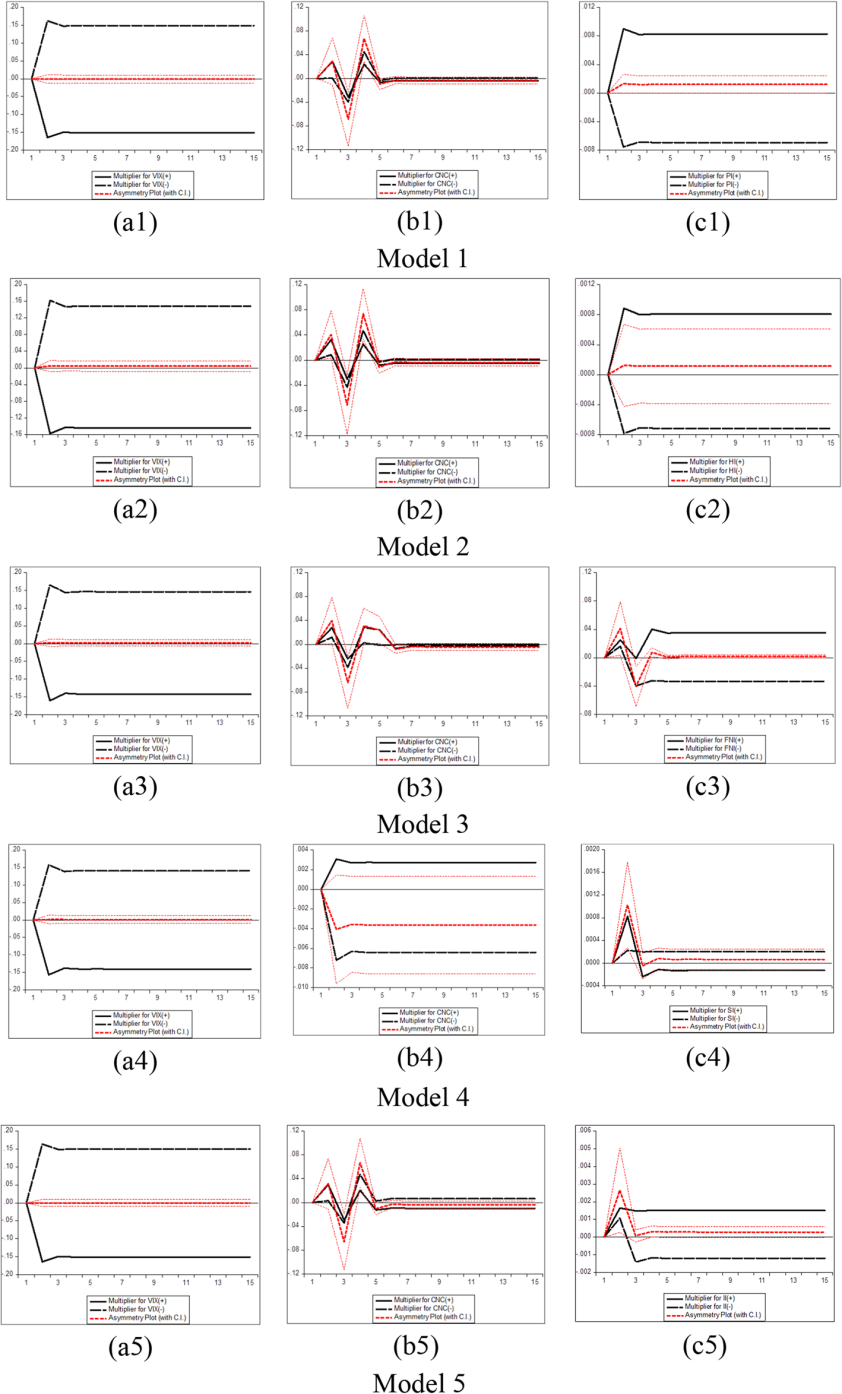

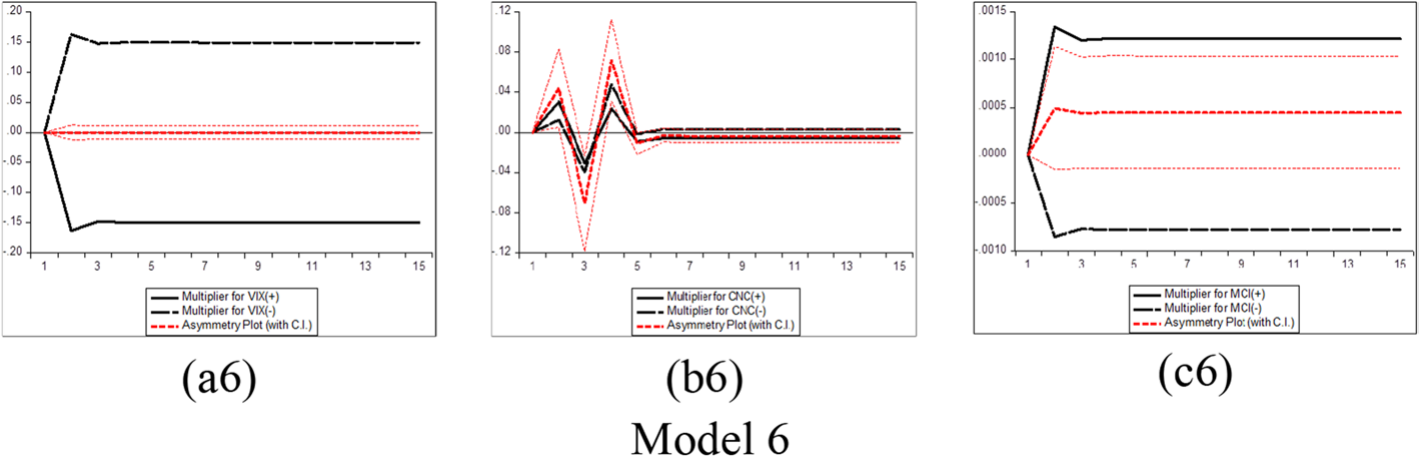
Asymmetric dynamic multipliers—Models 1–6: impact of positive and negative changes in VIX (a1–a6), daily number of new reported COVID-19 cases worldwide (b1–b6), each RavenPack coronavirus related indices (c1–c6) on daily changes of Bitcoin price. *Source* Authors’ own work. Notes: The horizontal axis shows the period (days) and the vertical axis the multiplier for positive (continuous black line) and negative (dashed black line) changes in VIX, daily number of new reported COVID-19 cases worldwide, each RavenPack coronavirus related indices and the asymmetry plot (dashed red line) with 95% bootstrap confidence interval based on 1000 replications. Variables’ description is provided in Table 4

Figure 4 presents the NARDL multipliers for models 1–6 that exhibit the impact of positive and negative changes of VIX, the daily number of newly reported COVID-19 cases worldwide, and each RavenPack coronavirus-related indices on daily changes of Bitcoin price, following González et al. (2021) and Gaies et al. (2021). The horizontal axis depicts the period in days, and the vertical axis reveals the multiplier for positive (continuous black line) and negative (dashed black line) changes in VIX, CNC, each RavenPack coronavirus-related indices, and the asymmetry (dashed red line) with 95% bootstrap confidence interval based on 1000 replications. If the 0 line is situated among the lower and upper bands, the asymmetric effects of the pandemic indices on Bitcoin are not significant at the 5% level.

The plots exhibit a specific asymmetric adjustment of RavenPack measures to the equilibrium due to positive and negative shocks in the long-run. Except for Coronavirus SI, the plots reveal the dominance of positive coronavirus indices shocks. In the fourth model, positive change in the SI initially dominates negative change, but afterward, negative shocks dominate positive change.

### Robustness check

To check the robustness of the quantitative outcomes, we re-estimate the NARDL models 1–6 by incorporating the daily number of newly reported COVID-19 deaths worldwide, following Iqbal et al. (2021) and Chen et al. (2022). Spiegel and Tookes (2021) argued the considerable differences in testing potential throughout time and territories, recommending centering on pandemic casualties rather than COVID-19 instances. Table 9 shows Bitcoin’s related short- and long-run asymmetric dynamic interactions with RavenPack coronavirus-related indices. The coefficient of the ECT indicates that disequilibrium in the Bitcoin returns from the short- to long-run is adjusted by 111% and 113% annually. Concerning pandemic indices, only the coefficients related to Coronavirus PI, Coronavirus HI, and Coronavirus FNI are statistically significant. The estimated values of the long-run coefficients ${\upbeta }^{+}$ equal 0.008382 ${(\mathrm{L}}_{\mathrm{PI}}^{+})$, 0.00099 $({\mathrm{L}}_{\mathrm{HI}}^{+})$, and 0.03054 $({\mathrm{L}}_{\mathrm{FNI}}^{+})$, while the coefficient ${\upbeta }^{-}$ equals 0.006814 ${(\mathrm{L}}_{\mathrm{PI}}^{-})$, 0.00072 ${(\mathrm{L}}_{\mathrm{HI}}^{-})$, and 0.028211 $({\mathrm{L}}_{\mathrm{FNI}}^{-})$. Unlike Vurur (2021), a 1% increase or decrease in PI, HI, and FNI increases Bitcoin returns by 0.008382% (0.006814%), 0.00099% (0.00072%), and 0.03054% (0.028211%), respectively. The outcomes support Béjaoui et al. (2021), who claimed that the pandemic fosters investing in Bitcoin. Panic in the equity market appears to be driving investors to invest in Bitcoin as one of the alternative assets (Anamika et al. 2021). Like Guégan and Renault (2021), the significant association between investor sentiment and Bitcoin returns is supported; hence, the empirical findings reinforce Chen et al. (2022), indicating that Bitcoin is recognized as a valuable alternative investment under uncertainty. Contrary to Burggraf et al. (2021), an increase in market volatility does not lead to a flight-to-safety phenomenon. Contrary to Choi and Shin (2022), Bitcoin is largely unaltered by COVID-19 panic shocks. Thus, including Bitcoin in the portfolio can mitigate the risk of a sudden decline in the value of investments triggered by exogenous shocks such as COVID-19 (Marobhe, 2022). Like Diaconaşu et al. (2022), we may notice that the Bitcoin market tends to mature. Besides, the Wald test results reveal that long-run asymmetry effects are confirmed merely in the case of PI. Concerning the long-term positive ${(\mathrm{L}}_{\mathrm{CND}}^{+})$ and negative ${(\mathrm{L}}_{\mathrm{CND}}^{-})$ shocks of the daily number of newly reported COVID-19 deaths worldwide, the influence on daily changes of Bitcoin price is negative in most cases, but the statistical significance is weak. Furthermore, similar to the outcomes from Table 8, both positive and negative shocks of VIX significantly negatively impact Bitcoin returns in the long-run.

**Table 9:** NARDL long-run and short-run estimates (daily number of new reported COVID-19 deaths worldwide included)

Model 7		Model 8		Model 9		Model 10		Model 11		Model 12	
$\mathrm{ECT}$	−1.116812*** (0.041939)	$\mathrm{ECT}$	−1.116606*** (0.042457)	$\mathrm{ECT}$	−1.139712*** (0.042209)	$\mathrm{ECT}$	−1.111821*** (0.042456)	$\mathrm{ECT}$	−1.122604*** (0.044665)	$\mathrm{ECT}$	−1.115852*** (0.042691)
*Long-run and short-run coefficients*											
Const	0.037845* (0.020121)	Const	0.036905 (0.023711)	Const	0.009091 (0.019562)	Const	0.005528 (0.0204)	Const	0.029139 (0.025177)	Const	0.013151 (0.018507)
${\mathrm{BTC}}_{\mathrm{t}-1}$	−1.116812*** (0.046306)	${\mathrm{BTC}}_{\mathrm{t}-1}$	−1.116606*** (0.046718)	${\mathrm{BTC}}_{\mathrm{t}-1}$	−1.139712*** (0.046655)	${\mathrm{BTC}}_{\mathrm{t}-1}$	−1.111821*** (0.04682)	${\mathrm{BTC}}_{\mathrm{t}-1}$	−1.122604*** (0.047313)	${\mathrm{BTC}}_{\mathrm{t}-1}$	−1.115852*** (0.047035)
${\mathrm{PI}}^{+}$	0.009361*** (0.0028)	${\mathrm{HI}}^{+}$	0.001105** (0.000509)	${\mathrm{FNI}}_{\mathrm{t}-1}^{+}$	0.034807*** (0.010502)	${\mathrm{SI}}_{\mathrm{t}-1}^{+}$	−0.000186 (0.000185)	${\mathrm{II}}^{+}$	0.00106 (0.000738)	${\mathrm{MCI}}^{+}$	0.000833 (0.000614)
${\mathrm{PI}}^{-}$	0.00761*** (0.002428)	${\mathrm{HI}}^{-}$	0.000804** (0.000367)	${\mathrm{FNI}}_{\mathrm{t}-1}^{-}$	0.032153*** (0.010253)	${\mathrm{SI}}^{-}$	−0.000142 (0.000123)	${\mathrm{II}}^{-}$	0.000773 (0.000625)	${\mathrm{MCI}}^{-}$	0.000551 (0.000574)
${\mathrm{CND}}^{+}$	−0.005202* (0.002734)	${\mathrm{CND}}^{+}$	−0.009405* (0.005348)	${\mathrm{CND}}^{+}$	−0.000274 (0.002605)	${\mathrm{CND}}^{+}$	0.000807 (0.003295)	${\mathrm{CND}}^{+}$	−0.005593 (0.005954)	${\mathrm{CND}}^{+}$	−0.004821 (0.005061)
${\mathrm{CND}}^{-}$	−0.002266 (0.002923)	${\mathrm{CND}}^{-}$	−0.008832 (0.00572)	${\mathrm{CND}}^{-}$	0.001366 (0.00347)	${\mathrm{CND}}^{-}$	0.001856 (0.003396)	${\mathrm{CND}}^{-}$	−0.003345 (0.005912)	${\mathrm{CND}}^{-}$	−0.003289 (0.004963)
${\mathrm{VIX}}^{+}$	−0.162725*** (0.023302)	${\mathrm{VIX}}^{+}$	−0.164996*** (0.023639)	${\mathrm{VIX}}^{+}$	−0.166015*** (0.023594)	${\mathrm{VIX}}^{+}$	−0.160503*** (0.024179)	${\mathrm{VIX}}_{\mathrm{t}-1}^{+}$	−0.204128*** (0.039936)	${\mathrm{VIX}}^{+}$	−0.163046*** (0.023716)
${\mathrm{VIX}}^{-}$	−0.159668*** (0.022556)	${\mathrm{VIX}}^{-}$	−0.162656*** (0.022708)	${\mathrm{VIX}}^{-}$	−0.163032*** (0.022781)	${\mathrm{VIX}}^{-}$	−0.163949*** (0.022803)	${\mathrm{VIX}}^{-}$	−0.202144*** (0.039239)	${\mathrm{VIX}}^{-}$	−0.162773*** (0.022816)
				$\Delta {\mathrm{FNI}}^{+}$	0.023703* (0.012186)	${\mathrm{\Delta SI}}^{+}$	0.000817* (0.000485)	$\Delta {\mathrm{VIX}}^{+}$	−0.143825*** (0.027046)		
				${\mathrm{\Delta FNI}}_{\mathrm{t}-1}^{+}$	−0.036948** (0.016707)						
				$\Delta {\mathrm{FNI}}^{-}$	−0.019016 (0.016799)						
*Long-run asymmetric effects*											
${L}_{PI}^{+}$	0.008382*** (0.002516)	${L}_{HI}^{+}$	0.00099** (0.000455)	${L}_{FNI}^{+}$	0.03054*** (0.009151)	${L}_{SI}^{+}$	−0.000167 (0.000167)	${L}_{II}^{+}$	0.000944 (0.000653)	${L}_{MCI}^{+}$	0.000747 (0.000547)
${L}_{PI}^{-}$	0.006814*** (0.002182)	${L}_{HI}^{-}$	0.00072** (0.000328)	${L}_{FNI}^{-}$	0.028211*** (0.008947)	${L}_{SI}^{-}$	−0.000128 (0.00011)	${L}_{II}^{-}$	0.000688 (0.000554)	${L}_{MCI}^{-}$	0.000494 (0.000513)
${L}_{CND}^{+}$	−0.004658* (0.002461)	${L}_{CND}^{+}$	−0.008423* (0.004792)	${L}_{CND}^{+}$	−0.00024 (0.002286)	${L}_{CND}^{+}$	0.000726 (0.002963)	${L}_{CND}^{+}$	−0.004982 (0.005291)	${L}_{CND}^{+}$	−0.00432 (0.004528)
${L}_{CND}^{-}$	−0.002029 (0.002625)	${L}_{CND}^{-}$	−0.00791 (0.005131)	${L}_{CND}^{-}$	0.001199 (0.00304)	${L}_{CND}^{-}$	0.001669 (0.00305)	${L}_{CND}^{-}$	−0.00298 (0.005263)	${L}_{CND}^{-}$	−0.002947 (0.004446)
${L}_{VIX}^{+}$	−0.145705*** (0.022474)	${L}_{VIX}^{+}$	−0.147766*** (0.022813)	${L}_{VIX}^{+}$	−0.145664*** (0.022364)	${L}_{VIX}^{+}$	−0.14436*** (0.023399)	${L}_{VIX}^{+}$	−0.181835*** (0.036636)	${L}_{VIX}^{+}$	−0.146118*** (0.022908)
${L}_{VIX}^{-}$	−0.142967*** (0.021791)	${L}_{VIX}^{-}$	−0.14567*** (0.021982)	${L}_{VIX}^{-}$	−0.143047*** (0.021637)	${L}_{VIX}^{-}$	−0.14746*** (0.022206)	${L}_{VIX}^{-}$	−0.180067*** (0.036004)	${L}_{VIX}^{-}$	−0.145873*** (0.022114)
Const	0.033886* (0.018053)	Const	0.033051 (0.021246)	Const	0.007977 (0.017155)	Const	0.004972 (0.018348)	Const	0.025956 (0.022367)	Const	0.011785 (0.016589)
*Long run asymmetry tests – Wald statistics*											
${\mathrm{W}}_{\mathrm{LR}}(\mathrm{PI})$	6.217237**	${\mathrm{W}}_{\mathrm{LR}}(\mathrm{HI})$	1.456352	${\mathrm{W}}_{\mathrm{LR}}(\mathrm{FNI})$	2.708718	${\mathrm{W}}_{\mathrm{LR}}(\mathrm{SI})$	0.408759	${\mathrm{W}}_{\mathrm{LR}}(\mathrm{II})$	2.901127	${\mathrm{W}}_{\mathrm{LR}}(\mathrm{MCI})$	0.595302
${\mathrm{W}}_{\mathrm{LR}}(\mathrm{CND})$	1.854015	${\mathrm{W}}_{\mathrm{LR}}(\mathrm{CND})$	0.076299	${\mathrm{W}}_{\mathrm{LR}}(\mathrm{CND})$	0.233893	${\mathrm{W}}_{\mathrm{LR}}(\mathrm{CND})$	0.288758	${\mathrm{W}}_{\mathrm{LR}}(\mathrm{CND})$	0.885304	${\mathrm{W}}_{\mathrm{LR}}(\mathrm{CND})$	0.512464
${\mathrm{W}}_{\mathrm{LR}}(\mathrm{VIX})$	0.388361	${\mathrm{W}}_{\mathrm{LR}}(\mathrm{VIX})$	0.165336	${\mathrm{W}}_{\mathrm{LR}}(\mathrm{VIX})$	0.511518	${\mathrm{W}}_{\mathrm{LR}}(\mathrm{VIX})$	0.472496	${\mathrm{W}}_{\mathrm{LR}}(\mathrm{VIX})$	0.187225	${\mathrm{W}}_{\mathrm{LR}}(\mathrm{VIX})$	0.002107
*Statistics and diagnostics*											
R-sq	0.164867	R−sq	0.151617	R−sq	0.188144	R−sq	0.152613	R−sq	0.150353	R−sq	0.144998
Adj R-sq	0.150253	Adj R−sq	0.13677	Adj R−sq	0.16759	Adj R−sq	0.13558	Adj R−sq	0.133275	Adj R−sq	0.130035
F-stat	11.28083***	F−stat	10.21215***	F−stat	9.153925***	F−stat	8.959883***	F−stat	8.803737***	F−stat	9.690706***
D-W stat	2.04387	D−W stat	2.055367	D−W stat	2.0712	D−W stat	2.039862	D−W stat	2.033129	D−W stat	2.050486
${\upchi }_{SC}^{2} (1)$	1.397077 [0.2379]	${\upchi }_{SC}^{2} (1)$	2.409233 [0.1214]	${\upchi }_{SC}^{2} (1)$	3.469847 [0.0632]	${\upchi }_{SC}^{2} (1)$	1.556456 [0.2129]	${\upchi }_{SC}^{2} (1)$	1.551059 [0.2137]	${\upchi }_{SC}^{2} (1)$	2.025632 [0.1554]
${\upchi }_{SC}^{2} (2)$	0.706231 [0.4941]	${\upchi }_{SC}^{2} (2)$	1.209319 [0.2995]	${\upchi }_{SC}^{2} (2)$	1.732613 [0.1782]	${\upchi }_{SC}^{2} (2)$	0.776286 [0.4608]	${\upchi }_{SC}^{2} (2)$	0.788258 [0.4553]	${\upchi }_{SC}^{2} (2)$	1.021123 [0.3611]
${\upchi }_{HET}^{2} (1)$	0.043841 [0.8343]	${\upchi }_{HET}^{2} (1)$	0.001695 [0.9672]	${\upchi }_{HET}^{2} (1)$	0.054961 [0.8148]	${\upchi }_{HET}^{2} (1)$	0.004986 [0.9437]	${\upchi }_{HET}^{2} (1)$	0.012057 [0.9126]	${\upchi }_{HET}^{2} (1)$	0.003922 [0.9501]
${\upchi }_{HET}^{2} (2)$	0.022385 [0.9779]	${\upchi }_{HET}^{2} (2)$	0.019461 [0.9807]	${\upchi }_{HET}^{2} (2)$	0.030659 [0.9698]	${\upchi }_{HET}^{2} (2)$	0.004753 [0.9953]	${\upchi }_{HET}^{2} (2)$	0.008388 [0.9916]	${\upchi }_{HET}^{2} (2)$	0.014115 [0.986]
${\upchi }_{NORM}^{2}$	1377.340 [0.0000]	${\upchi }_{NORM}^{2}$	1359.518 [0.0000]	${\upchi }_{NORM}^{2}$	1705.895 [0.0000]	${\upchi }_{NORM}^{2}$	1445.762 [0.0000]	${\upchi }_{NORM}^{2}$	1178.064 [0.0000]	${\upchi }_{NORM}^{2}$	1476.385 [0.0000]
${\upchi }_{\mathrm{RESET}}^{2}$	0.052934 [0.8182]	${\upchi }_{\mathrm{RESET}}^{2}$	0.08277 [0.7737]	${\upchi }_{\mathrm{RESET}}^{2}$	0.207103 [0.6493]	${\upchi }_{\mathrm{RESET}}^{2}$	0.069391 [0.7924]	${\upchi }_{\mathrm{RESET}}^{2}$	0.602375 [0.4381]	${\upchi }_{\mathrm{RESET}}^{2}$	0.309118 [0.5785]

*Source* Authors’ own computations. Notes: Superscripts ^*^, ^**^, ^***^ represent the significance at 10%, 5%, and 1% levels, respectively. The superscript + and – defines positive and negative partial sum. ${\mathrm{L}}^{+}$ and ${\mathrm{L}}^{-}$ are the computed long-run coefficients associated with positive and negative shocks, respectively. ${\mathrm{W}}_{\mathrm{LR}}$ denotes the Wald statistic for the long-run symmetry. ${\mathrm{W}}_{\mathrm{LR}}$ denotes the Wald statistic for the long-run symmetry, which tests the null hypothesis of ${\theta }^{+}$ =${\theta }^{-}$. ${\mathrm{\rm X}}_{\mathrm{SC}}^{2}$ denotes the Breusch-Godfrey Serial Correlation LM Test (first and second lag). ${\mathrm{\rm X}}_{\mathrm{HET}}^{2}$ denotes the Heteroskedasticity Test: ARCH (first and second lag). ${\mathrm{\rm X}}_{\mathrm{RESET}}^{2}$ denotes the Ramsey RESET Test of Misspecification. ${\mathrm{\rm X}}_{\mathrm{NORM}}^{2}$ denotes the Jarque–Bera test. The p-values of diagnostic tests are in []. Variables’ description is provided in Table 4

**Fig. 5:**
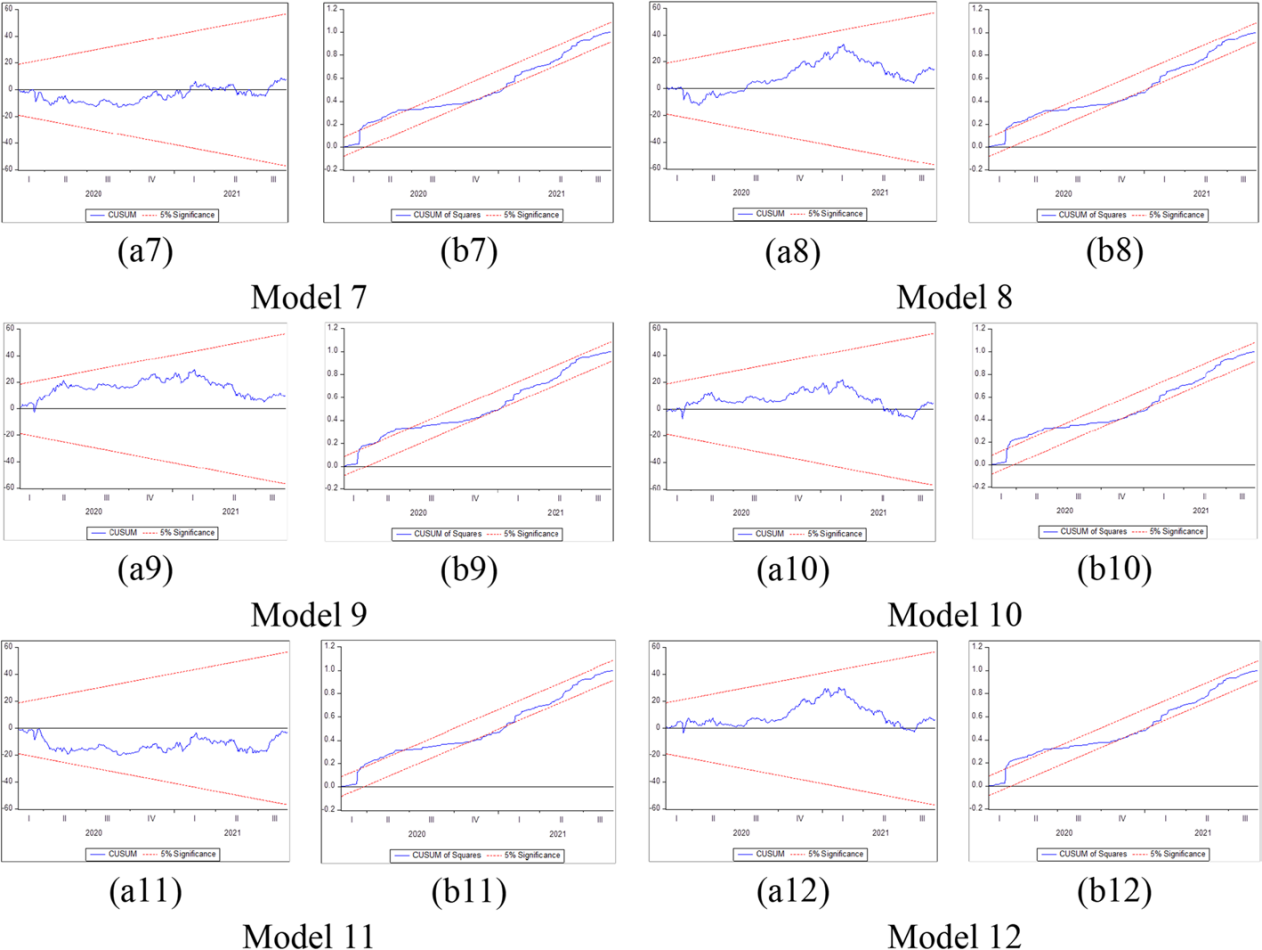
NARDL plots of cumulative sum of recursive residuals—CUSUM (a7–a12) and cumulative sum square of recursive residuals—CUSUMSQ (b7–b12) for Models 7–12. *Source* Authors’ own work. Notes: The blue line is the solid line while the red lines that bounded the blue line are the critical bounds at 0.5. Variables’ description is provided in Table 4

The diagnostic tests show that the estimated NARDL models 7–12 have no heteroskedasticity, serial correlation, or misspecification issues. Figure 5 illustrates that CUSUM and CUSUMSQ plots are within the 95% confidence level, denoting the stability of the estimated models.

**Fig. 6:**
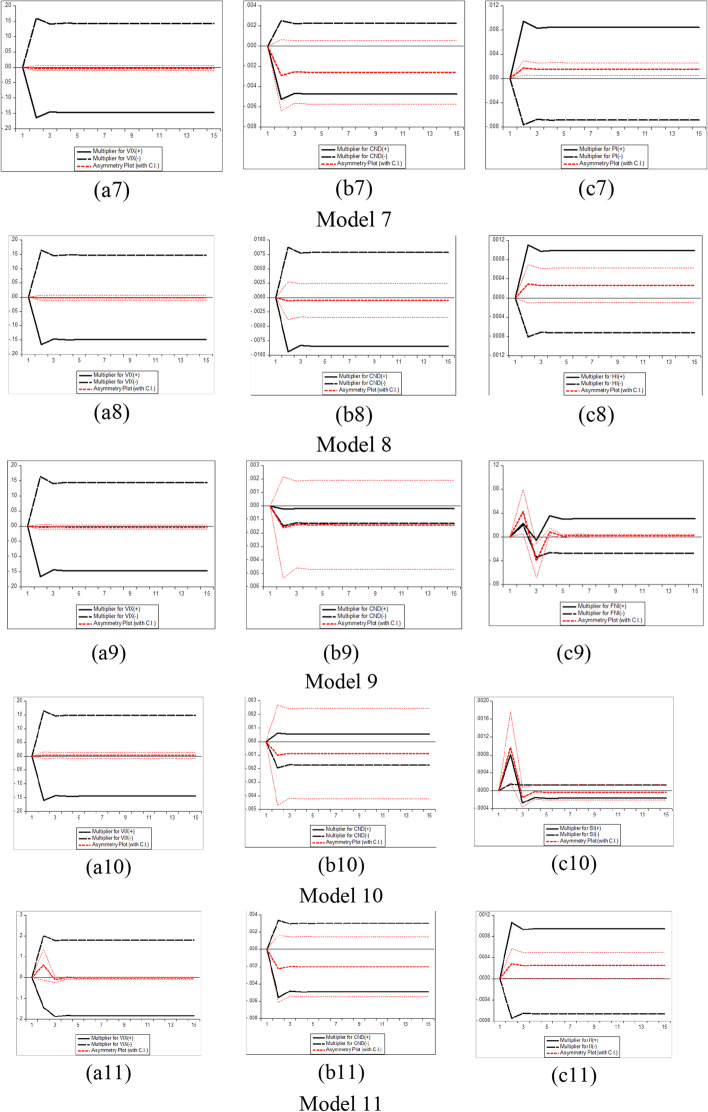

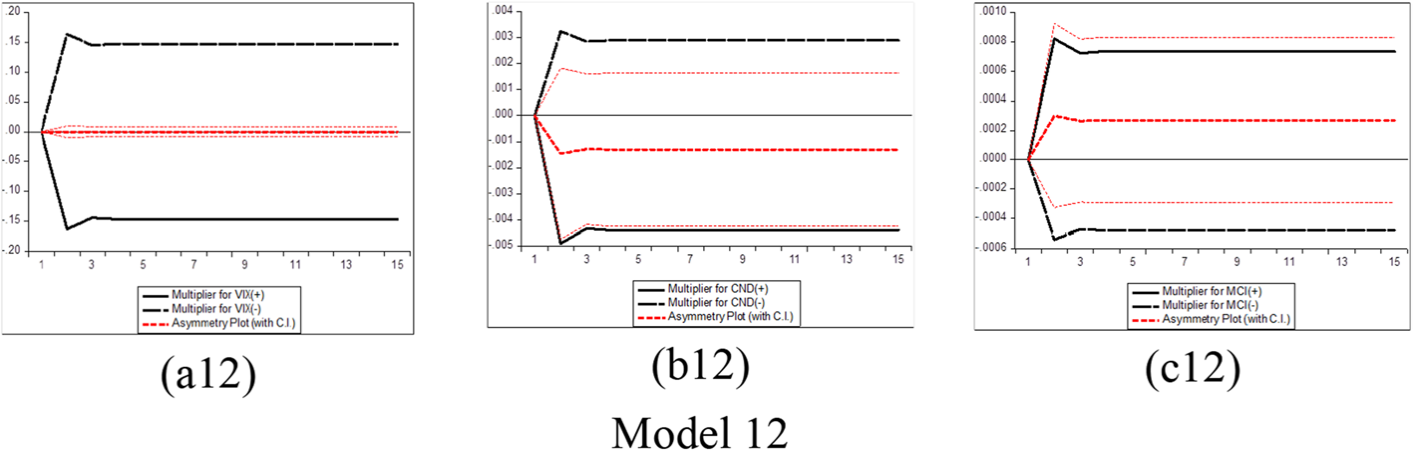
Asymmetric dynamic multipliers—Models 7- 12: impact of positive and negative changes in VIX (a7–a12), daily number of new reported COVID-19 deaths worldwide (b7–b12), each RavenPack coronavirus related indices (c7–c12) on daily changes of Bitcoin price. *Source* Authors’ own work. Notes: The horizontal axis shows the period (days) and the vertical axis the multiplier for positive (continuous black line) and negative (dashed black line) changes in VIX, daily number of new reported COVID-19 deaths worldwide, each RavenPack coronavirus related indices and the asymmetry plot (dashed red line) with 95% bootstrap confidence interval based on 1000 replications. Variables’ description is provided in Table 4

After the positive and negative variations influencing Bitcoin, the adjustment of asymmetries from initial long-term equilibrium to new long-term equilibrium can be regarded via the dynamic multipliers reported in Fig. 6 for models 7–12. The coronavirus indices reveal asymmetric adjustment patterns toward negative and positive shocks in the short and long-run. Similar to adjustment patterns reported in Fig. 4, among the RavenPack pandemic indices, Coronavirus SI exhibits an inverse relationship with Bitcoin returns. In contrast, direct relationships between all other coronavirus measures and Bitcoin occur in both the short- and long-run.

### Spectral causality analysis

**Fig. 7:**
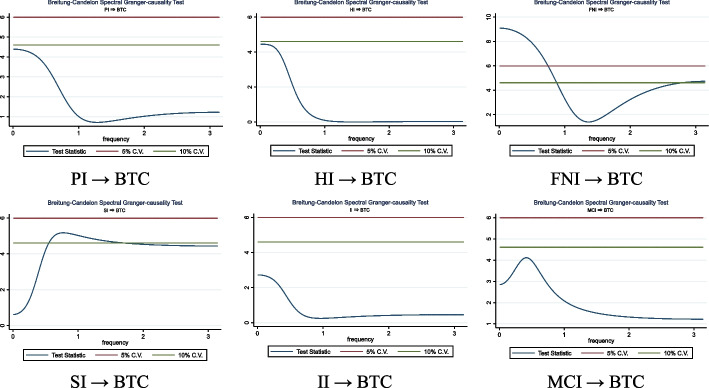
Plots of frequency domain Granger causality test from RavenPack coronavirus related indices to Bitcoin. *Source* Authors’ own work. Notes: The incidence of the connection between each RavenPack coronavirus related indices and daily changes of Bitcoin price is investigated at frequencies 2–3, 1–2, and 0–1. These frequencies show a short, medium, and long-term relationship. 0–1 is established as permanent causality, while 2–3 is recognized as temporary causality. The (red) upper line and the (brown) lower line represent statistically significant levels of 5 and 10%, respectively. The (blue) curves are used for statistical tests of various interval frequencies (0, π). Variables’ description is provided in Table 4

The frequency domain causality analysis outcomes from pandemic indices to Bitcoin are reported in Table 10, whereas Fig. 7 shows the associated plots. The horizontal red lines in Fig. 7 signify the relationship between the variables at a 5% significance level for all frequencies $(\omega )$ in the interval $(0, \pi )$. Frequency $(\omega )$ on the horizontal axis can be interpreted as a cycle or periodicity by $S=2\pi /\omega$, where *S* is the period. Hence, high frequencies match short periods, and short frequencies relate to long periods. Unlike prior studies that used Twitter Happiness sentiment and found no Granger causality (Naeem et al. 2020, 2021b), the results support a long-term causal relationship running from Coronavirus FNI to Bitcoin, as well as a medium-term causal relationship from Coronavirus SI to Bitcoin. The outcomes support Mokni et al. (2022), who found an asymmetric causality only throughout the pandemic phase, and Polat et al. (2022), who reported that fear caused Bitcoin’s return in the post-COVID era. Likewise, the findings align with Guégan and Renault (2021), who noticed that investor sentiment Granger causes Bitcoin returns. Furthermore, the outcomes are consistent with Zhu et al. (2021), suggesting that investor attention is a significant factor in the Bitcoin market. Additionally, Banerjee et al. (2022) proved a unidirectional causal relationship between COVID-19 news sentiment and cryptocurrency returns. However, the rest of RavenPack's coronavirus-related indices do not cause Bitcoin at any frequency range.

**Table 10:** Results of frequency domain causality test from RavenPack coronavirus-related indices to Bitcoin

	Long term		Medium term		Short term	
${\omega }_{i}$	0.01	0.05	1	1.5	2	2.5
PI → BTC	4.3928	4.3879	1.0100	0.7792	1.0201	1.1636
HI → BTC	4.4489	4.4461	0.0962	0.0008	0.0147	0.0272
FNI → BTC	9.0938^**^	9.0839^**^	3.2947	1.5765	3.2324	4.3087
SI → BTC	0.6145	0.6374	5.0260^*^	4.6845^*^	4.5306	4.4632
II → BTC	2.7229	2.7147	0.2455	0.3452	0.4133	0.4456
MCI → BTC	2.8498	2.8828	2.0840	1.5089	1.3221	1.2481

*Source*: Authors’ own computations. Notes: Superscripts *, **, *** represent the significance at 10%, 5%, and 1% levels, respectively. Variables’ description is provided in Table 4

**Fig. 8:**
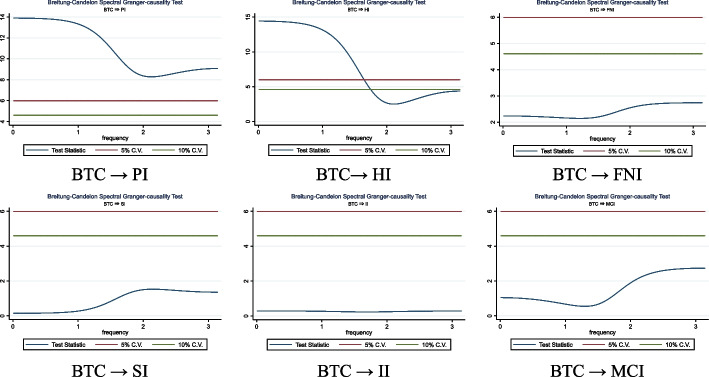
Plots of frequency domain Granger causality test from Bitcoin to RavenPack coronavirus-related indices. *Source* Authors’ own work. Notes: The incidence of the connection between daily changes of Bitcoin price and each RavenPack coronavirus-related indices is investigated at frequencies 2–3, 1–2, and 0–1. These frequencies show a short, medium, and long-term relationship. 0–1 is established as permanent causality, while 2–3 is recognized as temporary causality. The (red) upper line and the (brown) lower line represent statistically significant levels of 5% and 10%, respectively. The (blue) curves are used for statistical tests of various interval frequencies (0, π). Variables’ description is provided in Table 4

Table 11 shows the Breitung–Candelon spectral Granger causality test results from Bitcoin to pandemic indices, and Fig. 8 exhibits the related plots. The outcomes support that Bitcoin Granger causes Coronavirus PI for all frequencies. Significant Granger causality is also found from Bitcoin to Coronavirus HI in the medium and long-run.

**Table 11:** Results of frequency domain causality test from Bitcoin to RavenPack coronavirus-related indices

${\omega }_{i}$	Long term		Medium term		Short term	
	0.01	0.05	1	1.5	2	2.5
BTC → PI	13.9038***	13.9034***	13.3386***	11.0315***	8.3672**	8.6330**
BTC → HI	14.4251***	14.4240***	13.1558***	8.2573**	2.6569	3.3568
BTC → FNI	2.2370	2.2368	2.1579	2.1994	2.5456	2.7131
BTC → SI	0.1473	0.1474	0.2717	0.8303	1.5014	1.4583
BTC → II	0.2886	0.2886	0.2674	0.2351	0.2425	0.2716
BTC → MCI	1.0456	1.0447	0.6717	0.6640	1.8917	2.5952

*Source* Authors’ own computations. Notes: Superscripts *, **, *** represent the significance at 10%, 5%, and 1% levels, respectively. Variables’ description is provided in Table 4

## Concluding remarks and policy implications

This paper examined whether daily changes in Bitcoin price react to COVID-19 pandemic news. The asymmetric volatility examination through EGARCH (1,1) model exhibited that adverse and optimistic news have the same effect, hence the FOMO behavior not being supported. By employing the NARDL framework, we reinforced prior literature (Rognone et al. 2020) and found positive and negative shocks in RavenPack coronavirus-related indices (Panic, Hype, Fake News, and Infodemic) stimulate Bitcoin returns; hence, during market instability, Bitcoin can withstand foreign shocks and act as a hedge. Additionally, we could argue that the cryptocurrency market seems resilient to the endless frictions brought on by the COVID-19 pandemic. Furthermore, we conclude that cryptocurrencies could be a crucial component of portfolio diversification. Moreover, in line with prior studies that used Twitter-based uncertainty measures (Wu et al. 2021b; Aharon et al. 2020), the outcomes of the Breitung–Candelon spectral Granger causality test reveals a one-way causality running from Coronavirus FNI and SI to Bitcoin returns, whereas Bitcoin price Granger cause Coronavirus PI and HI. Accordingly, Bitcoin might influence future investor behavior in the markets for virtual currencies.

Since the risk portfolios of worldwide investors and portfolio managers may be severely affected by the pandemic, acknowledging the conduct of digital currencies throughout times of intense tension, such as a COVID-19 pandemic and informed trading, is necessary. Therefore, investors can rely on RavenPack coronavirus-related indices as a significant driver of Bitcoin return and shape trading approaches accordingly. Understanding the connection between Bitcoin and panic can provide investors with insights for portfolio optimization or risk mitigation to deal with digital assets’ price volatility. Therefore, investors should consider incorporating cryptocurrencies for their portfolios’ optimization and diversification and use crypto assets for expenditures and fund transfers. Likewise, this research may be helpful to regulators and governments in developing policies to alleviate this market, lessen its significant instability, and boost investor trust. Authorities can assess the emotion-driven cryptocurrency crisis and take appropriate measures. As such, the government should enact appropriate legislation to guide the marketplace. Furthermore, authorities should supervise unethical strategies of cryptocurrency trading to assist economies in achieving economic security and investment gains.

The results of this study have several limitations. First, it is imperative to emphasize that our data set covers only Bitcoin returns. Future research could implement a broader range of cryptocurrencies, such as Litecoin, Ethereum, Tether, and Ripple, to investigate the effects of COVID-19 pandemic news on their returns. Our study is also limited to indices provided by a data and analytics vendor, such as RavenPack. Therefore, future research should construct investor sentiment indices based on Google search terms or Twitter feeds. Another future study could divide the period to investigate the impact of pandemic news on the cryptocurrency market during each COVID-19 wave. Finally, more regions where Bitcoin is used in transactions could be covered.

## Data Availability

The data regarding daily changes of Bitcoin price (BTC/USD – Bitcoin US Dollar) and daily change of Chicago Board Options Exchange (CBOE) volatility index was extracted from Investing.com. The data towards Coronavirus Panic Index, Coronavirus Hype Index, Coronavirus Fake News Index, Coronavirus Sentiment Index, Coronavirus Infodemic Index, Coronavirus Media Coverage Index was provided by RavenPack. The data concerning daily number of new reported COVID-19 cases worldwide and daily number of new reported COVID-19 deaths worldwide was extracted from Our World in Data. The datasets used and/or analyzed during the current study are available from the corresponding author on reasonable request.

## Appendix 1: Correlation matrix

**Table:**

Variables	BTC	PI	HI	FNI	SI	II	MCI	CNC	CND	VIX
BTC	1									
PI	0.123655 (2.513950) [0.0123]	1								
HI	0.107594 (2.183297) [0.0296]	0.841293 (31.39673) [0.0000]	1							
FNI	0.141377 (2.881121) [0.0042]	0.630175 (16.37358) [0.0000]	0.613236 (15.66221) [0.0000]	1						
SI	−0.037591 (−0.758905) [0.4483]	−0.406196 (−8.967844) [0.0000]	−0.212660 (−4.390693) [0.0000]	−0.275183 (−5.774562) [0.0000]	1					
II	0.080079 (1.620730) [0.1059]	0.563126 (13.74761) [0.0000]	0.868108 (35.28258) [0.0000]	0.435786 (9.767957) [0.0000]	0.137075 (2.791730) [0.0055]	1				
MCI	0.078619 (1.591006) [0.1124]	0.541095 (12.98060) [0.0000]	0.786199 (25.66611) [0.0000]	0.452513 (10.23721) [0.0000]	0.205522 (4.236697) [0.0000]	0.927261 (49.96246) [0.0000]	1			
CNC	0.029534 (0.596090) [0.5514]	−0.380820 (−8.308830) [0.0000]	−0.317501 (−6.754854) [0.0000]	−0.185570 (−3.809918) [0.0002]	0.395259 (8.680932) [0.0000]	−0.040817 (−0.824143) [0.4103]	0.182236 (3.739092) [0.0002]	1		
CND	0.027990 (0.564900) [0.5725]	−0.278242 (−5.844108) [0.0000]	−0.108485 (−2.201588) [0.0283]	−0.111876 (−2.271274) [0.0237]	0.331712 (7.093672) [0.0000]	0.173603 (3.556304) [0.0004]	0.359786 (7.779350) [0.0000]	0.865146 (34.80147) [0.0000]	1	
VIX	−0.355755 (−7.679478) [0.0000]	−0.043960 (−0.887727) [0.3752]	−0.091778 (−1.859390) [0.0637]	−0.063497 (−1.283587) [0.2000]	−0.034042 (−0.687177) [0.4924]	−0.145795 (−2.973061) [0.0031]	−0.135570 (−2.760501) [0.0060]	−0.079184 (−1.602514) [0.1098]	−0.118101 (−2.399382) [0.0169]	1

*Source*: Authors’ own computations. Notes: Statistics in () exhibit t-statistic. Figures in [] shows Probability | t |= 0. Variables’ description is provided in Table 4

## Appendix 2: Rolling correlations among RavenPack coronavirus related indices, daily number of novel pandemic cases and fatalities worldwide

*Source* Authors’ own work. Notes: The window width for the rolling correlation is specified at 14 days. Variables’ description is provided in Table 4.

## Appendix 3 EGARCH (1,1) estimation results for BTC.

**Table:**

Dependent Variable: BTC
Method: ML ARCH—Student's t distribution (BFGS / Marquardt steps)
Sample: 1/23/2020 9/01/2021
Included observations: 409
Convergence achieved after 56 iterations
Coefficient covariance computed using outer product of gradients
Presample variance: backcast (parameter = 0.7)
LOG(GARCH) = C(2) + C(3)*ABS(RESID(-1)/@SQRT(GARCH(-1))) + C(4)
*RESID(-1)/@SQRT(GARCH(-1)) + C(5)*LOG(GARCH(-1))

Variable	Coefficient	Std. Error	z-Statistic	Prob
C	0.003805	0.001630	2.334675	0.0196
*Variance Equation*				
C(2)	−0.275439	0.131835	-2.089274	0.0367
C(3)	0.178966	0.056358	3.175516	0.0015
C(4)	0.007022	0.037809	0.185708	0.8527
C(5)	0.975685	0.018051	54.05028	0.0000
T-DIST. DOF	3.665338	0.596147	6.148382	0.0000
R-squared	−0.001190	Mean dependent var		0.005396
Adjusted R-squared	−0.001190	S.D. dependent var		0.046169
S.E. of regression	0.046197	Akaike info criterion		-3.599932
Sum squared resid	0.870722	Schwarz criterion		-3.541051
Log likelihood	742.1861	Hannan−Quinn criter		-3.576635
Durbin-Watson stat	2.284140			

*Source* Authors’ own computations. Notes: Variables’ description is provided in Table 4

## Appendix 4: EGARCH (1,1) estimation results for VIX

**Table:**

Dependent Variable: VIX
Method: ML ARCH—Student's t distribution (BFGS / Marquardt steps)
Sample: 1/23/2020 9/01/2021
Included observations: 409
Convergence achieved after 58 iterations
Coefficient covariance computed using outer product of gradients
Presample variance: backcast (parameter = 0.7)
LOG(GARCH) = C(2) + C(3)*ABS(RESID(-1)/@SQRT(GARCH(-1))) + C(4)
*RESID(-1)/@SQRT(GARCH(-1)) + C(5)*LOG(GARCH(-1))

Variable	Coefficient	Std. Error	z-Statistic	Prob
C	−0.004652	0.003136	-1.483295	0.1380
*Variance Equation*				
C(2)	−0.103987	0.030672	-3.390260	0.0007
C(3)	−0.054849	0.034820	-1.575195	0.1152
C(4)	0.248480	0.058882	4.219943	0.0000
C(5)	0.978304	0.006471	151.1869	0.0000
T-DIST. DOF	3.289368	0.503823	6.528820	0.0000
R-squared	−0.009460	Mean dependent var		0.004694
Adjusted R-squared	−0.009460	S.D. dependent var		0.096205
S.E. of regression	0.096659	Akaike info criterion		-2.294186
Sum squared resid	3.811906	Schwarz criterion		-2.235305
Log likelihood	475.1610	Hannan-Quinn criter		-2.270889
Durbin-Watson stat	2.250316			

*Source* Authors’ own computations. Notes: Variables’ description is provided in Table 4

## Appendix 5: Unit root tests without structural break

**Table:**

Variables	Level			First difference		
	Intercept	Trend & Intercept	Without Trend & Intercept	Intercept	Trend & Intercept	Without Trend & Intercept
Augmented Dickey-Fuller (ADF) unit root test						
BTC	−23.3183^***^	−23.3046^***^	−22.9733^***^	−12.3983^***^	−12.3828^***^	−12.4142^***^
PI	−2.4313	−3.5029^**^	−0.8805	−10.9065^***^	−10.9349^***^	−10.92^***^
HI	−2.2718	−3.4114^*^	−0.6307	−6.59^***^	−6.7135^***^	−6.5991^***^
FNI	−5.4963^***^	−5.919^***^	−1.4313	−15.8406^***^	−15.8421^***^	−15.8598^***^
SI	−2.5676	−2.4272	−2.1437^**^	−15.0561^***^	−15.0784^***^	−15.0709^***^
II	−3.334^**^	−4.2269^***^	−0.103	−5.9017^***^	−6.1673^***^	−5.9009^***^
MCI	−4.463^***^	−4.805^***^	0.1025	−5.9614^***^	−6.1128^***^	−5.9656^***^
CNC	−1.7964	−3.5092^**^	−0.2738	−4.524^***^	−4.5251^***^	−4.4493^***^
CND	−1.9367	−2.5732	0.3844	−13.8935^***^	−13.9742^***^	−13.8083^***^
VIX	−23.1004^***^	−23.1667^***^	−23.0652^***^	−13.0221^***^	−13.0047^***^	−13.0389^***^
*Phillips-Perron (PP) unit root test*						
BTC	−23.2346^***^	−23.2229^***^	−22.8089^***^	−333.8464^***^	−332.7252^***^	−333.1452^***^
PI	−7.6604^***^	−9.2185^***^	−1.8205^*^	−40.9492^***^	−41.2412^***^	−41.0032^***^
HI	−2.8282^*^	−3.7526^**^	−0.5925	−26.9138^***^	−27.2029^***^	−26.9304^***^
FNI	−12.6538^***^	−12.9709^***^	−3.3545^***^	−52.6608^***^	−52.9635^***^	−52.7215^***^
SI	−3.4058^**^	−3.6036^**^	−2.5101^**^	−29.3143^***^	−56.469^***^	−27.5883^***^
II	−3.7799^***^	−4.0972^***^	−0.0226	−32.0588^***^	−33.8911^***^	−31.9084^***^
MCI	−5.2236^***^	−5.2389^***^	0.4232	−25.7852^***^	−26.3721^***^	−25.7087^***^
CNC	−2.2708	−5.6518^***^	−0.3036	−31.9399^***^	−31.9068^***^	−31.485^***^
CND	−4.6461^***^	−9.0937^***^	−0.8469	−50.3275^***^	−50.9265^***^	−49.5784^***^
VIX	−23.0469^***^	−23.1582^***^	−22.9739^***^	−371.2423^***^	−394.2407^***^	−355.8698^***^
*Kwiatkowski-Phillips-Schmidt-Shin (KPSS) unit root test*						
BTC	0.0946	0.0717		0.1361	0.1358^*^	
PI	0.897^***^	0.0606		0.0995	0.0567	
HI	0.7604^***^	0.1408^*^		0.2696	0.1067	
FNI	0.4478^*^	0.0791		0.0705	0.0379	
SI	0.8363^***^	0.3795^***^		0.2488	0.1262^*^	
II	0.3709^*^	0.2537^***^		0.4983^**^	0.1696^**^	
MCI	0.2715	0.2762^***^		0.6052^**^	0.1915^**^	
CNC	1.962^***^	0.247^***^		0.0396	0.0357	
CND	1.7298^***^	0.2842^***^		0.0673	0.0354	
VIX	0.1803	0.0837		0.0417	0.0413	

*Source* Authors’ own computations. Notes: Values are reported for Dickey-Fuller (ADF), Phillips-Perron (PP), and Kwiatkowski-Phillips-Schmidt-Shin (KPSS) unit root test methods. Null hypothesis is non-stationary for ADF&PP, and stationary for KPSS. Lag Length based on SIC. Superscripts *, **, *** represent the significance at 10%, 5%, and 1% levels, respectively. Variables’ description is provided in Table 4

## Appendix 6: Unit root test with structural breaks

**Table:**

Variables	Zivot-Andrews (ZA) unit root test					
			Level			
	Intercept	Break point	Trend	Break point	Trend & Intercept	Break point
BTC	−23.59259^***^	2/22/2021	−23.33515	1/07/2021	−23.7118^***^	5/10/2021
PI	−3.360543^*^	5/12/2020	−3.129722	6/07/2021	−3.145402	5/25/2021
HI	−3.629078	8/04/2020	−3.52505	6/07/2021	−3.457124	6/02/2021
FNI	−4.852416^**^	5/05/2020	−4.741216	6/03/2021	−	−
SI	−6.091661^***^	5/18/2020	−5.546161^***^	7/06/2020	−6.102909^*^	5/18/2020
II	−5.846642	8/13/2020	−5.496813	6/07/2021	−5.425831	6/07/2021
MCI	−5.389047^*^	9/09/2020	−4.996208	6/07/2021	−4.988391^***^	5/17/2021
CNC	−3.355426^***^	5/10/2021	−1.72165	10/29/2020	−3.140505^***^	5/13/2021
CND	−2.616653^***^	6/02/2021	−2.092303^***^	12/30/2020	−3.195966^***^	11/03/2020
VIX	−23.38269	4/22/2020	−	−	−23.59767	5/12/2020
*First difference*						
BTC	−15.04517	2/09/2021	−14.3471	5/13/2021	−15.04401	5/20/2021
PI	−11.04359	4/28/2020	−	−	−11.28913	6/12/2020
HI	−7.136702	4/28/2020	−7.226811	5/13/2020	−7.38726^***^	6/05/2020
FNI	−15.91408^*^	4/28/2020	−15.91691	5/01/2020	−15.95095	6/11/2020
SI	−15.24377^**^	12/28/2020	−15.09312	4/30/2020	−15.24878	6/30/2020
II	−9.658423^**^	5/04/2020	−9.496702^**^	6/02/2020	−9.696578^*^	5/12/2020
MCI	−6.537531	4/30/2020	−7.888586	5/12/2020	−8.052636^***^	6/09/2020
CNC	−10.10256^***^	1/11/2021	−9.733051	6/04/2021	−10.64056^***^	5/03/2021
CND	−14.4765^***^	1/25/2021	−14.00867	6/04/2021	−14.51875^***^	1/25/2021
VIX	−15.16655	1/28/2021	−15.12925	6/02/2021	−15.14689^**^	6/12/2020

*Source* Authors’ own computations. Notes: Superscripts *, **, *** represent the significance at 10%, 5%, and 1% levels, respectively. Variables’ description is provided in Table 4.

## Abbreviations

COVID-19: Coronavirus disease 2019
SARS-CoV-2: Severe acute respiratory syndrome coronavirus 2
FOMO: Fear of missing out
EGARCH: Exponential generalized autoregressive conditional heteroskedasticity
NARDL: Nonlinear autoregressive distributed lag
